# Impact of aging on gut-lung-adipose tissue interactions and lipid metabolism during influenza infection in mice

**DOI:** 10.1038/s41598-025-21363-1

**Published:** 2025-10-27

**Authors:** Gemma Bogard, Kassem Makki, Patricia Brito-Rodrigues, Jian Tan, Olivier Molendi-Coste, Johanna Barthelemy, Amandine Descat, Fabrice Bouilloux, Cécile Lecoeur, Corinne Grangette, Cyril Robil, Jean-François Goossens, Philippe Gosset, Laurence Macia, François Trottein, Isabelle Wolowczuk

**Affiliations:** 1 Univ. Lille, Centre National de la Recherche Scientifique (CNRS), Institut National de la Santé et de la Recherche Médicale (Inserm), Centre Hospitalier Universitaire de Lille (CHU Lille), Institut Pasteur de Lille, U1019 – UMR9017 – CIIL – Center for Infection and Immunity of Lille, F-59000 Lille, France; 2 Laboratoire CarMeN, Inserm U1060, INRAe UMR1397, Université Claude Bernard Lyon1, Pierre Bénite, F-69310 Villeurbanne, France; 3 Charles Perkins Centre, the University of Sydney, Sydney, Australia; 4 School of Medical Sciences, Faculty of Medicine and Health, The University of Sydney, Sydney, Australia; 5 Univ. Lille, CNRS, Inserm, CHU Lille, Institut Pasteur de Lille, US 41 – UAR 2014 – PLBS, F-59000 Lille, France; 6 Univ. Lille, CHU Lille,, ULR 7365 – GRITA – Groupe de Recherche sur les formes Injectables et les Technologies Associées, F-59000 Lille, France; 7 Biomnigene, F-25000 Besançon, France

**Keywords:** Microbiology, Virology, Influenza virus

## Abstract

Influenza remains a major threat to human health, especially for the elderly. Aging leads to substantial changes to lung function, gut microbiota, and white adipose tissue (WAT)—a key endocrine organ regulating energy balance and lipid metabolism. In the current study, we performed a multi-omics analysis to investigate how influenza impacts the gut-lung-adipose tissue axis differently with age at days 2, 4, 7, 14, and 28 post-infection (dpi). Compared to young-adult mice, aged mice experienced worse disease outcomes following infection, along with distinct WAT alterations, including impaired browning, heightened inflammation, and reduced innate immune cell recruitment. Age-related differences were also evidenced in infection-driven shifts in gut microbiota. *Akkermansia* levels rose only in young mice from 4 dpi, while *Faecalibaculum* and *Muribaculum* expanded exclusively in aged mice at 7 dpi, the only timepoint at which their abundance correlated with lung pathology. Serum metabolomics at 7 dpi also revealed age-dependent metabolic responses to infection. Compared to their non-infected counterparts, young mice had lower levels of p-Cresol-sulfate and Indoxyl-sulfate alongside higher triglycerides, whereas aged mice showed disrupted glycerophospholipid metabolism. By pinpointing specific gut bacteria as potential probiotics and identifying lipid pathways associated with disease progression, these findings could lead to the development of targeted, age-specific strategies to mitigate influenza severity in the elderly.

**Supplementary Information:**

The online version contains supplementary material available at 10.1038/s41598-025-21363-1.

## Introduction

Influenza A virus (IAV) infections remain a major global public health challenge, driving recurrent seasonal outbreaks and occasional pandemics, and significantly contributing to severe respiratory diseases^1^. Worldwide, IAV infections are estimated to cause 3 to 5 million cases of severe illness and approximately 290,000 to 650,000 respiratory deaths each year^2^. Influenza-related morbidity and mortality disproportionately affect several at-risk populations, including individuals with obesity and those aged 65 and over^3,4^. Notably, more than 90% of annual influenza-related deaths occur among the elderly^5^.

Chronological aging is associated with a marked decline in both innate and adaptive immunity, a process known as immunosenescence^6^. This age-related immune dysfunction weakens the host’s ability to defend against respiratory intruders and to generate an effective response following vaccination^4^. Additionally, inflammaging, a state of chronic low-grade inflammation, is a hallmark of the aging immune system^7^. Advanced age also leads to progressive impairment of pulmonary functions, including reduced lung elasticity, weakened respiratory muscles, diminished lung capacity, and impaired mucociliary clearance of inhaled pathogens^8,9^. Other factors, such as comorbidities and nutritional deficiencies, further compromise the ability of older adults to respond to and recover from influenza^10,11^. Given the significant role of the gut-lung axis in respiratory viral infections^12,13^, the age-related changes in gut microbiota may also be critical in this context. Aging is associated with shifts in gut microbiota composition, diversity, and functional activity^14,15^, including a reduction in beneficial bacteria like *Bifidobacteria*^16,17^. These age-related changes in gut microbiota can increase gut permeability, promote inflammation, and impair immune responses^18^. Previous studies, including our own, have shown that influenza infection induces gut dysbiosis, which favors respiratory and enteric bacterial superinfections, resulting in more severe disease outcomes^13,19–22^. However, the combined impact of advanced age and virus-induced gut dysbiosis on disease severity remains unknown.

Aging also affects the white adipose tissue (WAT), an endocrine organ that regulates systemic energy metabolism^23^, notably through its interactions with the gut microbiota as part of the gut-adipose tissue axis^24^. These age-related changes include an increase in visceral WAT (VAT) mass at the expense of subcutaneous WAT (SCAT) mass, a shift in adipokine profiles that promote a proinflammatory state, and dysregulation of resident innate and adaptive immune cells^25–29^. Compelling evidence indicates that WAT can be targeted by pathogens^30^, including respiratory viruses such as severe acute respiratory syndrome coronavirus 2 (SARS-CoV-2)^31,32^ and IAVs^33,34^, and can also generate effective immune responses against infections^35,36^. In our recent findings with young-adult mice (designated as “young”), we observed that influenza infection altered lipid metabolism in both SCAT and VAT and induced thermogenic browning features exclusively in SCAT^34^. Noticeably, hematopoietic cells (CD45^+^ cells) carrying viral RNA and antigens were detected in the WAT of infected mice, with higher frequencies in SCAT compared to VAT^34^. However, the consequences of influenza on WAT in aged mice remain unexplored.

We hypothesized that age-related changes in the lungs, gut microbiota, and WAT—collectively forming the gut-lung-adipose tissue axis—contribute to the weakened response to and increased severity of influenza in older adults. To investigate this, we conducted a comprehensive analysis of infection-induced effects on the lungs, WAT, gut microbiota, and serum metabolome in both young and aged mice. Our findings identified distinct age-related alterations in WAT, gut microbiota, and metabolite profiles in infected mice, some of which correlated with lung disease severity, potentially contributing to the heightened severity of influenza outcomes in the elderly.

## Results

### Young mice and aged mice exhibit distinct early and late outcomes following influenza

**Fig. 1:**
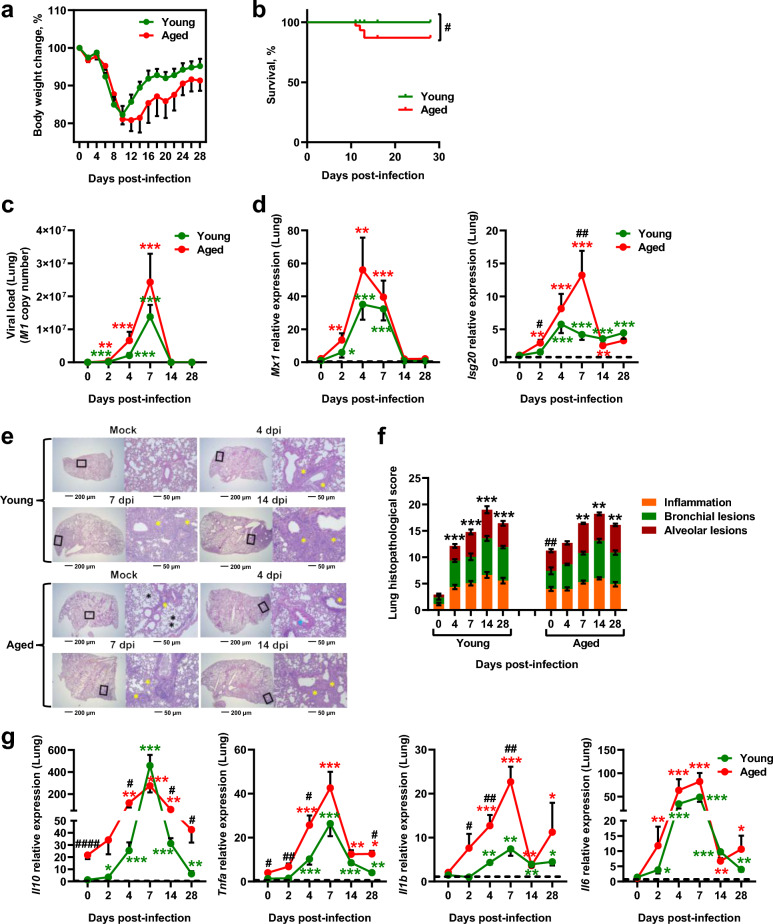
Influenza infection is more severe in aged mice than in young mice. Mice aged 2 months (“young”) and 18 months (“aged”) were intranasally treated with H3N2 influenza A virus (IAV) or PBS (Mock) (the study design is presented Supplementary Fig. 1a, experiment 1). **(a)** Percentage body weight change (% from initial body weight) during infection. Data are expressed as mean ± SEM, n=35 mice per age group. **(b)** Survival curves during infection. All infected young mice survived through 28 days post-infection (dpi). From infected aged mice, 1 died at 11 dpi, 1 died at 12 dpi and 1 died at 13 dpi. **(c)** Viral load in lung tissues at 0, 2, 4, 7, 14 and 28 dpi, n=7 per age group at each time point, except for aged mice at 28 dpi (n=4). **(d)** mRNA expression levels of *Mx1* (encoding MX Dynamin Like GTPase1) and *Isg20* (encoding interferon-stimulated gene 20) in lung tissues at 0, 2, 4, 7, 14 and 28 dpi, n=7 per age group at each time point, except for aged mice at 28 dpi (n=4). **(e)** Representative photomicrographs of lung tissues (H&E staining) at 0 (Mock), 4, 7 and 14 dpi. Framed regions were zoomed. Yellow stars: inflammation areas, Blue stars: bronchial lesions, Black stars: alveolar lesions. **(f)** Histopathological scoring of lung tissues at 0, 4, 7, 14 and 28 dpi, n=7 per age group at each time point, except for aged mice at 28 dpi (n=4). The sum of the subscores (inflammation, bronchial lesions and alveolar lesions) is shown. **(g)** mRNA expression levels of *Il10*, *Tnfa*, *Il1b*, and *Il6* in lung tissues at 0, 2, 4, 7, 14 and 28 dpi, n=7 per age group at each time point, except for aged mice at 28 dpi (n=4). For **d** and **g**: Relative expression is presented as 2^-ΔΔCT^. Data were normalized to *Gapdh* housekeeping gene (encoding glyceraldehyde 3-phosphate dehydrogenase) expression levels, and expressed relative to the expression obtained in samples from mock-treated young mice. For **c**, **d**, **f**, and **g**: Data are expressed as mean ± SEM. Data were analyzed using a two-sided Mann-Whitney test, with ^#^ indicating *P* values for aged vs. young group comparisons (^#^*P* < 0.05, ^##^*P* < 0.01, ^###^*P* < 0.001) and ^*^indicating *P* values for mock vs. infected group comparisons (^*^P < 0.05, ^**^P < 0.01, ^***^P < 0.0001). *P* < 0.05 was considered statistically significant.

Young mice (2 months of age, n=35) and aged mice (18 months of age, n=35) were infected with a dose of IAV (H3N2 subtype) that is sublethal for young mice, and tissue samples were collected at various days post-infection (dpi) (see study design Supplementary Fig. 1a, experiment 1). Prior to infection, aged mice had significantly higher body weight than young mice (Supplementary Fig. 1b), as previously reported^37^. Although both age groups showed comparable weight loss between 1 and 10 dpi, young mice gradually regained weight from 10 dpi. In contrast, aged mice continued losing weight until 12 dpi, after which they began to recover, albeit at a significantly slower rate than younger mice (Fig. 1a). Additionally, while all the 35 young mice survived through day 28 after infection, some aged mice did not (from the 35 infected aged mice, one died at 11 dpi, one died at 12 dpi and 1 died at 13 dpi) (Fig. 1b). In both age groups, pulmonary viral RNA levels increased from 2 dpi, peaked at 7 dpi, and became undetectable thereafter (Fig. 1c); however, aged mice showed overall higher levels, though this difference was not statistically significant. Infection induced a significant upregulation of pulmonary interferon-stimulated genes (ISGs), such as *Mx1* and *Isg20* (indicators of viral replication), as early as 2 dpi in both young and aged mice. Notably, *Isg20* expression was significantly higher in the lungs of aged mice compared to young mice at 2 and 7 dpi (Fig. 1d). Next, to evaluate and compare lung pathology and inflammation in young and aged mice following infection, histological and gene expression analyses were performed. Lung sections were stained with H&E to assess histopathological changes. Consistent with previous findings^38^, aging alone (i.e., without infection) was associated with structural deterioration of the alveolar walls, as evidenced by enlarged airspaces and blunted alveolar septa, as well as with inflammatory cell infiltration around bronchi and blood vessels (Fig. 1e, Mock panels). Following infection, both young and aged mice exhibited signs of lung inflammation at 4 dpi, with aged mice displaying more frequent bronchial epithelial hyperplasia and consolidated interstitial pneumoniae. By 7 dpi and 14 dpi, both age groups showed bronchoepithelial hyperplasia, bronchointerstitial pneumonia, and mixed inflammatory cell infiltrates in perivascular and intra-alveolar areas (Fig. 1e). Semiquantitative scoring of the histological sections (subscores: inflammation, bronchial lesions, and alveolar lesions) confirmed that aging alone significantly increased baseline lung inflammation and tissue damage (Fig. 1f and Supplementary Fig. 1c, 0 dpi panels). Although total histopathological scores did not differ significantly between age groups at 4, 7, 14, or 28 dpi (Fig. 1f), aged mice exhibited more alveolar lesions than young mice at 4 dpi (Supplementary Fig. 1c). At 28 dpi, both groups still showed areas of residual inflammation, with young mice exhibiting heightened peri-bronchial inflammation, whereas emphysematous-like lesions were more frequently observed in aged mice (Supplementary Fig. 1d). However, this observation should be interpreted cautiously given the small number of aged mice assessed at 28 dpi (n=4). Baseline expression levels of the inflammation-related genes *Il10* and *Tnfa* were elevated in aged mice (Fig. 1 g, 0 dpi panels), indicating a pre-existing inflammatory state. After infection, the expression levels of *Il10*, *Tnfa*, *Il1b* and *Il6* increased significantly in both young and aged mice at 4 dpi, peaking at 7 dpi. Notably, the infection-induced expression of *Il10*, *Tnfa*, and *Il1b* was significantly higher in aged mice compared to young mice (Fig. 1g).

Collectively, these results suggest that aged mice develop greater pulmonary inflammation following influenza infection compared to younger mice, which may contribute to their higher morbimortality.

### Influenza induces depot-specific changes in WAT that differ between young and aged mice

**Fig. 2:**
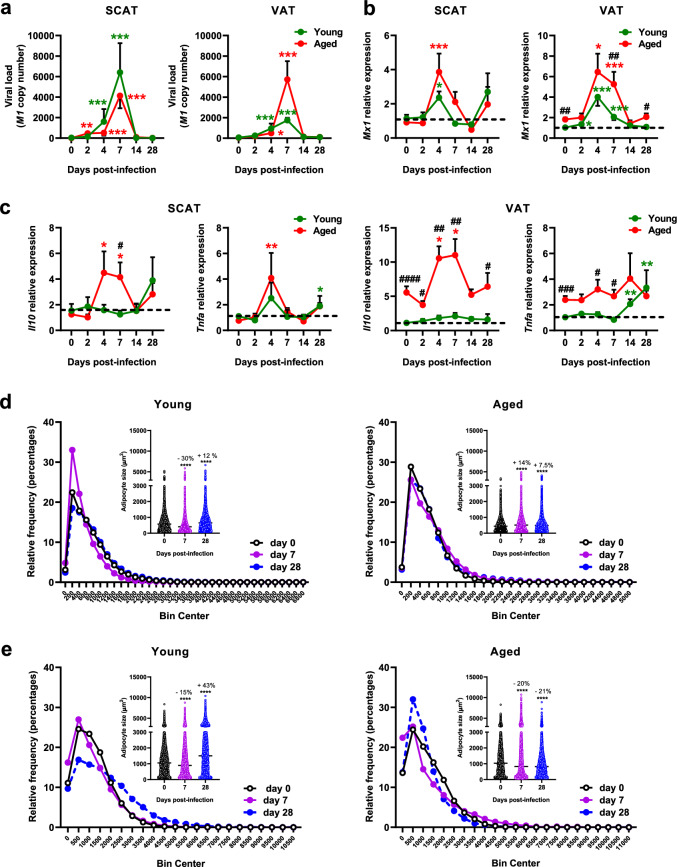
Influenza-infection-induced changes in the white adipose tissue differentiate aged mice from young mice. **(a)** Viral load in subcutaneous and visceral white adipose tissues (SCAT and VAT, respectively) at 0, 2, 4, 7, 14, and 28 dpi. **(b)** mRNA expression levels of *Mx1* in SCAT and VAT at 0, 2, 4, 7, 14, and 28 dpi. **(c)** mRNA expression levels of *Il10* and *Tnfa* in SCAT (left) and VAT (right) at 0, 2, 4, 7, 14, and 28 dpi. **(d)** Relative frequency plots (%) of adipocyte size distribution in the SCAT from young (left) and aged (right) mice at 0, 7 and 28 dpi. Adipocyte counts were binned by size (bin intervals=200 μm^2^) and normalized to the total number of counted adipocytes. Superplots display individual adipocyte sizes and mean values (µm^2^; insert). **(e)** Relative frequency plots (%) of adipocyte size distribution in the VAT from young (left) and aged (right) mice at 0, 7 and 28 dpi. Adipocyte counts were binned by size (bin intervals=500 μm^2^) and normalized to the total number of counted adipocytes. Superplots display individual adipocyte sizes and mean values (µm^2^; insert). For **a**, **b** and **c**: Data are expressed as mean ± SEM, n=7 per age group at each time point, except for aged mice at 28 dpi (n=4) For **b** and **c**: Relative expression is presented as 2^-ΔΔCT^. Data were normalized to *Eef2* housekeeping gene (encoding eukaryotic translation elongation factor 2) expression levels, and expressed relative to the expression obtained in samples from mock-treated young mice. For **d** and **e**: for SCAT: n=4 animals per group except n=3 for aged mice at 28 dpi, and a mean (range) of 2213 (1261-3192) adipocytes per tissue sample were analyzed. For VAT: n=7 animals per group except n=3 for aged mice at 28 dpi, and a mean (range) of 1212 (596 - 1558) adipocytes per tissue sample were analyzed. Groups were compared using a two- sided Mann-Whitney test, with ^#^ indicating *P* values for age group comparisons (^#^*P* < 0.05, ^##^*P* < 0.01, ^###^*P* < 0.001, ^####^*P* < 0.0001), and ^*^ indicating *P* values for mock vs. infected group comparisons (^*^P < 0.05, ^**^P < 0.01, ^***^P < 0.001). *P* < 0.05 was considered statistically significant.

Bidirectional interactions between the lungs and WAT have been reported during respiratory infections. For instance, IAV infection rewires WAT energy metabolism^34^, and the loss of fat cells has been linked to an increased burden of *Mycobacterium tuberculosis* in the lungs, worsening pulmonary pathology^39^. We thus compared the effects of infection on WAT’s metabolism, inflammation, and immune cell composition between young and aged mice. Quantification of viral RNA levels showed a time-dependent accumulation in both SCAT and VAT depots, peaking at 7 dpi before declining. Although aged mice exhibited higher viral RNA levels in VAT at 7 dpi compared to young mice, the difference was not statistically significant (Fig. 2a). Expression of antiviral response-related genes (*Mx1*, *Rig1* and *Mda5*) increased significantly in SCAT and VAT in both age groups (Fig. 2b and Supplementary Fig. 2a). In SCAT, *Mx1* expression peaked at 4 dpi and returned to baseline in both young and aged mice. In VAT, *Mx1* expression increased at both 4 and 7 dpi across age groups. Notably, at 7 dpi, *Mx1* expression in aged VAT was significantly higher than in young mice. Before infection, aging was associated with increased expression of inflammation-related markers (*Il10*, *Tnfa*, and *Il1b*) in VAT, but not in SCAT (Fig. 2c and Supplementary Fig. 2b). Notably, infection drove age- and depot-specific inflammatory responses in WAT. In SCAT of aged mice, infection induced an increase in *Il10* levels at 4 and 7 dpi, *Tnfa* at 4 dpi*,* and both *Il1b* and *Il6* at 7 dpi. Conversely, in young mice, infection led to reduced *Il1b* levels at 7 dpi. In VAT, *Tnfa* and *Il1b* levels increased in young mice at 14 and 28 dpi, while *Il10* was upregulated in aged mice at 4 and 7 dpi, and *Il6* increased earlier in aged mice than in young mice (4, 7 and 14 dpi vs. 28 dpi) (Fig. 2c and Supplementary Fig. 2b).

**Fig. 3:**
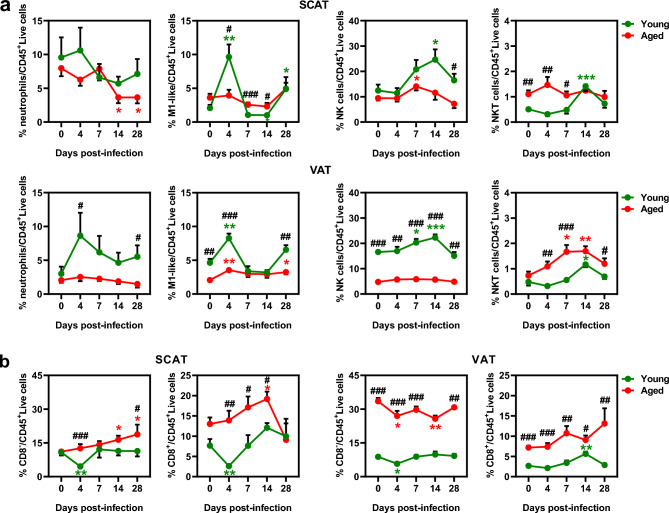
Influenza-infection-induced changes in adipose immune cells composition differentiate aged mice from young mice. Immunophenotypic characterization of adipose innate and adaptive immune cells in young and aged mice at 0, 4, 7, 14 and 28 dpi (n=7 per age group at each time point, except for aged mice at 28 dpi (n=4)). **(a)** Innate immune cell (neutrophils, M1-like macrophages, NK cells, NKT cells) frequencies among CD45^+^ Live cells in SCAT (above) and VAT (below). **(b)** CD8^-^ and CD8^+^ T cell frequencies among CD45^+^ Live cells in SCAT (left) and VAT (right). Data are expressed as mean ± SEM. For **a**: Neutrophils were identified as Live-Dead^-^/CD45^+^CD11b^+^Ly6G^hi^ cells, M1-like macrophages as Live- Dead^-^/CD45^+^CD11b^+^F4/80^+^CD11c^+^CD206^-^ cells, NK cells as Live-Dead^-^/CD45^+^NK1.1^+^CD3ε^-^ cells, and NKT cells as Live-Dead^-^/CD45^+^NK1.1^+^ CD3ε^+^ cells. Groups were compared using a two-sided Mann-Whitney test, with ^#^ indicating *P* values for age group comparisons (^#^*P* < 0.05, ^##^*P* < 0.01, ^###^*P* < 0.001), and ^*^ indicating *P* values for mock vs. infected group comparisons (^*^P < 0.05, ^**^P < 0.01, ^***^P < 0.001). *P* < 0.05 was considered statistically significant. The gating strategy is presented Supplementary Fig. 10.

The age-dependent effects of infection on adipocyte size and distribution in SCAT and VAT, were assessed using quantitative histomorphometry (Supplementary Table 1 and Supplementary Table 2). Before infection, aged mice had smaller adipocytes in SCAT compared to young mice (Supplementary Fig. 2c), suggesting enhanced lipolysis and reduced lipogenesis, as supported by increased *Lipe* expression and decreased *Glut4* expression in aged mice (Supplementary Fig. 2d). Infection transiently reduced adipocyte size in young SCAT at 7 dpi, an effect that disappeared by 28 dpi (Fig. 2d). The reduction in adipocyte size observed in young mice, but not in aged mice, was likely due to infection-induced SCAT browning, as previously reported^34^. Indeed, the expression of *Ucp1*, which encodes mitochondrial uncoupling protein 1—a marker of thermogenic brown adipose tissue^40^—was increased as early as 2 dpi in young SCAT, whereas it remained unchanged in aged SCAT (Supplementary Fig. 2e). Consistently, histological analysis of SCAT from young mice during infection revealed the emergence of small, multilocular adipocytes featuring brown-like adipocytes^41^ at 2 dpi, with peak accumulation observed at 7 dpi (Supplementary Fig. 2f). In contrast, SCAT from aged mice showed no evidence of brown-like adipocytes at any point during the infection (data not shown). This suggests that aged mice exhibit impaired SCAT browning in response to infection. In VAT, the impact of infection on adipocyte size varied with age: at 28 dpi, young VAT had larger adipocytes, while aged VAT had smaller ones (Fig. 2e). We next characterized immune cell populations in the stromal vascular fractions of SCAT and VAT, as well as in lungs, in an independent infection experiment (see study design Supplementary Fig. 1a, experiment 2). Before infection, aging influenced innate and adaptive immune cells differently across these depots. Aged SCAT had fewer dendritic cells but unchanged macrophages and natural killer (NK) cells, whereas aged VAT had fewer macrophages and NK cells but stable dendritic cells (Supplementary Fig. 3a). Additionally, aged VAT had more T cells (CD3, CD4, CD8) than young VAT, as shown by RT-qPCR and phenotypic analysis, whereas aged SCAT did not show significant changes (Supplementary Fig. 3b). Notably, infection altered both innate and adaptive immune cell compositions of SCAT and VAT, with clear distinctions between young and aged mice (Fig. 3, Table 1 and Table 2). For innate immune cells (Fig. 3a), young mice showed increases in M1-like macrophages (4 dpi), NK cells (14 dpi), and natural killer T (NKT) cells (14 dpi) in both SCAT and VAT following infection. In contrast, aged mice showed limited innate immune changes, which varied by depot: in SCAT, NK cells increased (7 dpi) and neutrophils decreased (14 and 28 dpi), while VAT had increased M1-like macrophages (4 dpi) and NKT cells (7 and 14 dpi). The adaptive immune response to infection was more pronounced in aged mice, especially in SCAT (Fig. 3b). In SCAT, CD8^-^ T cells, which primarily represent CD4^+^ T cells but may also include TCR γδ T cells, along with CD8⁺ T cells, decreased at 4 dpi in young mice. In aged mice, however, these cells showed a delayed increase—CD8^-^ T cells at 14 and 28 dpi, and CD8⁺ T cells at 14 dpi. In VAT, CD8^-^ T-cell levels decreased at 4 dpi in young mice, with a similar reduction observed in aged mice at both 4 and 14 dpi. In contrast, CD8⁺ T-cell levels increased at 14 dpi in young mice but remained unchanged in aged mice.

**Table 1:** Frequency of immune cells among CD45^+^ live cells in the SCAT from young mice and aged mice during influenza infection.

	**Young**					**Aged**				
	**Mock**	**4 dpi**	**7 dpi**	**14 dpi**	**28 dpi**	**mock**	**4 dpi**	**7 dpi**	**14 dpi**	**28 dpi**
Neutrophils	9.6	10.6	6.6	5.7	7.1	8	6.3	7.9	3.7^*^	3.7^*^
Mφ	21.4	44.3^*^	21.9	18.4	26.6	29.5	26.6^##^	21.9	19.5	37
M1-like Mφ	2.1	9.6^**^	1.1	_1_*	4.9^*^	3.6^#^	3.9^#^	_2.6_###	2.3^#^	5
M2-like Mφ	5.9	3.8	1.2	0.9	4.7	12.6	_4.6_***	_4.3_**, #	_5.1_*, #	9.3
M0-like Mφ	13.2	30.3^**^	19.4	16.3	17.4	13.2	17.6^##^	14.6	11.8	18.6
Dendritic cells	3.7	2.8	2.4^*^	2.8	3.2	2.5^#^	2.7^*^	_3.9_**,##	2.6	2.1
NK cells	12.5	11.4	20.8	24.6^*^	16.5	9.5	9.4	14.1^*^	11.6	7.2^#^
NKT cells	0.5	0.3	0.5	_1.4_***	0.7	1.1^##^	1.5^##^	1.1^#^	1.2	1
B cells	30.1	12.9	15	17.7	15.1	12.6	8.8	_5.1_***, #	16	14.3
T cells	18.5	7.2^**^	19.9	23.5	21.5	24.2	30.3^###^	31.4	35.7^*, #^	29.1
CD8^-^ T cells	10.8	4.5^**^	9	11.4	9.2	11.1	12.6^###^	14.2^#^	16.4^*^	18.8^*, #^
CD8^+^ T cells	7.7	2.6^**^	7.7	12.1	10	13	17.6^###^	17.1^#^	19.2^*, #^	14.6

Immune cells were characterized by flow cytometry (refer to Gating strategy Supplementary Fig. 10), n=7 per group at each time point, except for aged mice at 28 dpi (n=4). Groups were compared using a two-sided Mann-Whitney test, with ^#^ indicating *P* values for age group comparisons (^#^*P* < 0.05, ^##^*P* < 0.01, ^###^*P* < 0.001), and ^*^ indicating *P* values for mock vs. infected group comparisons (^*^P < 0.05, ^**^P < 0.01, ^***^P < 0.001). *P* < 0.05 was considered statistically significant. Mφ : macrophages.

**Table 2:** Frequency of immune cells among CD45^+^ live cells in the VAT from young mice and aged mice during influenza infection.

	**Young**					**Aged**				
	**Mock**	**4 dpi**	**7 dpi**	**14 dpi**	**28 dpi**	**mock**	**4 dpi**	**7 dpi**	**14 dpi**	**28 dpi**
Neutrophils	3	8.6	6.2	4.7	5.5	2.1	2.5^#^	2.3	1.9	1.5
Mφ	38.8	44.2	30.8	31	42.8	27.9^#^	27.4	24.9	36^*^	29
M1-like Mφ	4.7	8.3^**^	3.4	3.2	6.6	2.1^##^	3.5^**^	3	2.9	3.3^*^
M2-like Mφ	7.5	3.3^*^	3.1	7	7.5	9.2	5.1^**^	_7.5_**,##	8.5	6.8
M0-like Mφ	26.1	32.3	24	20.3	28.2	16.2^#^	18.4^#^	14^#^	24.2^*^	18.5
Dendritic cells	4.1	2.6^*^	4.63	3.8	4.7	3.1	1.6^*^	3.8	2.7	3.2
NK cells	16.6	17	20.3^*^	22.3^***^	15.1	_4.8_###	5.7^##^	_5.8_###	_5.7_###	4.9^##^
NKT cells	0.5	0.3	0.6	1.2^*^	0.7	0.7	1.1^##^	_1.7_*,###	1.7^**^	1.2^#^
B cells	5.9	2.4^*^	3.4	4.4	4.6	6.7	5.7^##^	4	7.4^#^	5.3
T cells	11.6	_8_*	12.3	15.6	12.1	40.8^###^	34.5^###^	40.5^###^	_34.9_*,###	_44_##
CD8^-^ T cells	8.8	5.8^*^	8.9	9.8	9.2	33.5^###^	27^*^	29.7	25.8^**^	30.8
CD8^+^ T cells	2.7	2.1	3.4	5.6^**^	2.9	_7.2_###	_7.4_###	10.7^##^	9.1^#^	13.1^##^

Immune cells were characterized by flow cytometry (refer to Gating strategy Supplementary Fig. 10), n=7 per group at each time point, except for aged mice at 28 dpi (n=4). Groups were compared using a two-sided Mann-Whitney test, with # indicating *P* values for the age group comparisons (^#^*P* < 0.05, ^##^*P* < 0.01, ^###^*P* < 0.001), and ^*^ indicating *P* values for mock vs. infected group comparisons (^*^P < 0.05, ^**^P < 0.01, ^***^P < 0.001). *P* < 0.05 was considered statistically significant. Mφ : macrophages.

Interestingly, the immune cell composition in the lungs (see gating strategy Supplementary Fig. 4a) showed patterns similar to those observed in VAT. Aging alone resulted in a decrease in NK cells and an increase in NKT and CD8^+^ T cells in the lungs (Supplementary Fig. 4b, and Supplementary Table 3), and infection led to an increase in NKT cells in the lungs (Supplementary Fig. 4c, and Supplementary Table 3), as seen in VAT. However, infection had contrasting effects on CD8^-^ T cells, increasing their numbers in the lungs (Supplementary Fig. 4d, and Supplementary Table 3) but decreasing them in VAT.

These findings underscore age-dependency in the effects of influenza infection on WAT depots. In aged mice, SCAT browning was impaired, and inflammatory marker expression was increased in both SCAT and VAT, suggesting a heightened inflammatory response following infection. Additionally, aged mice showed alterations in the innate immune response in both SCAT and VAT, along with an enhanced T-cell response specifically in SCAT.

### Age-dependent correlations between lung and WAT responses to influenza

**Fig. 4:**
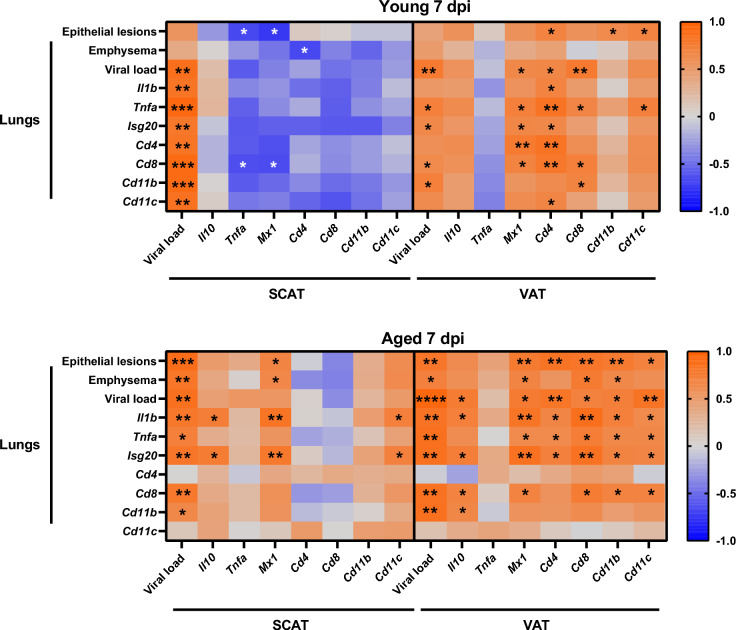
Correlations at 7 dpi between lung pathology, viral load, inflammation and immune cells, and SCAT and VAT viral load, inflammation and immune cells. Heatmap representing Spearman’s correlations between lung epithelial lesions, emphysema, viral mRNA levels, *Il1b*, *Tnfa*, *Isg20*, *Cd4*, *Cd8*, *Cd11b* and *Cd11c* mRNA levels, and SCAT and VAT viral mRNA levels, *Il10*, *Tnfa*, *Mx1, Cd4*, *Cd8*, *Cd11b* and *Cd11c* mRNA levels at 7 dpi, in young and aged mice, n=5 per group. Blue and red colors stand for inverse and positive correlations, respectively. The symbols ^*^ on plots represent significant correlations (^*^P < 0.05, ^**^P < 0.01, ^***^P < 0.001, ^****^P < 0.0001). *P* < 0.05 was considered statistically significant.

To further explore the relationship between infection-induced changes in lungs and WAT at 7 dpi, which corresponds to the acute phase of infection, we performed pairwise Spearman correlation analyses (Fig. 4). In young mice, lung pathology inversely correlated with SCAT levels of *Tnfa*, *Mx1* (epithelial lesions), and *Cd4* (emphysema). Additionally, lung viral load, inflammation, and immune cells positively correlated with SCAT viral load. In VAT, positive correlations were observed between *Cd4*, *Cd11b*, and *Cd11c* expression levels and lung epithelial lesions. Furthermore, there were positive correlations between lung viral load and VAT viral load, and between lung inflammation and antiviral response, and VAT levels of *Mx1*, *Cd4*, and *Cd8*. In stark contrast, aged mice exhibited numerous positive correlations between lung pathology markers (epithelial lesions and emphysema), lung inflammation, and lung antiviral response with WAT features, predominantly in VAT.

These findings reveal age-dependent differences in the association between lung pathology and infection-induced alterations in WAT at 7 dpi. In young mice, lung pathology correlates negatively with changes in SCAT and shows limited positive correlations with VAT’s changes. In contrast, aged mice display multiple positive correlations between lung pathology and changes in both fat depots, particularly VAT. These patterns highlight distinct relationship between WAT depots and lung pathology across age groups.

### Influenza induces distinct changes in gut microbiota composition and function in young mice and aged mice

The connection between WAT and gut microbiota, known as the gut-adipose tissue axis, is well-established^24^. Notably, gut microbiota is an important factor modulating WAT browning triggered by cold exposure or intermittent fasting^42–44^, and contributes to the regulation of WAT immune milieu and inflammation^45^. Besides, the gut-lung axis affects respiratory infection outcomes^13^, with gut dysbiosis observed in influenza-infected mice^19–22^, although research on aged mice remains limited^46^. To further investigate the gut-lung-adipose tissue axis, we compared the effects of infection on gut microbiota diversity, composition, and function in young vs. aged mice using 16S rRNA gene sequencing.

Before infection, aged mice exhibited significantly lower gut bacterial α-diversity compared to young mice, as indicated by significantly reduced operational taxonomic unit (OTU) richness and Shannon’s diversity index (Supplementary Fig. 5a), consistent with previous findings^15^. Additionally, aged mice showed an increased abundance of the Verrucomicrobiota phylum (formerly Verrucomicrobia^47^) and a trend (*P*=0.053) toward a lower abundance of the Pseudomonadota phylum (formerly Proteobacteria^47^), with other major phyla remaining similar across age groups (Supplementary Fig. 5b). At the family level, aged mice had more Lachnospiraceae, Akkermansiaceae, and UBA932, and fewer Oscillospiraceae, Rikenellaceae, Butyricicoccaceae, and RUG350 compared to young mice (Supplementary Fig. 5c). At the genus level, aged mice had higher levels of *Akkermansia*, *Acetatifactor*, and *Kineothrix*, and lower levels of *Lawsonibacter*, *Faecalibaculum*, and *Alistipes* (Supplementary Fig. 5d). To gain deeper mechanistic insights, PICRUSt2 was used to conduct a predictive metagenomic analysis. This analysis identified eight Kyoto Encyclopedia of Genes and Genomes (KEGG) pathways with significant differential enrichment between young and aged mice (*P* < 0.05). These pathways were primarily involved in the biosynthesis of cofactors, carriers, and vitamins (such as heme, tetrahydrofolate, and pyridoxal-phosphate biosynthesis) as well as in energy metabolism (such as mitochondrial respiration) (Supplementary Table 4). All predicted metabolic pathways showed reduced activity in aged mice. The reduced diversity and functional capacity of the gut microbiome observed in aged mice was associated with elevated blood levels of the proinflammatory cytokines IL-6 and IL-23 (Supplementary Fig. 5e), supporting the notion that age-related gut dysbiosis is linked to systemic inflammation^48^.

**Fig. 5:**
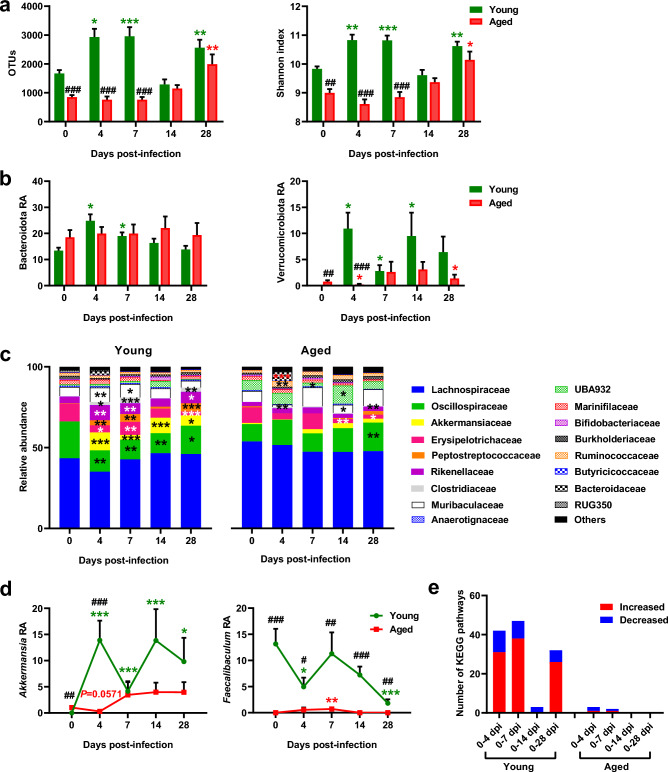
Influenza infection alters gut microbiota composition and function more in young mice than in aged mice. **(a)** Alpha diversity metrics. Species richness (OTUs) and species diversity (Shannon diversity index) were compared between the gut microbiota of mock-treated and infected young and aged mice at 4, 7, 14 and 28 dpi. **(b)** Relative abundances (RA) of *Bacteroidota* and *Verrucomicrobiota* phyla in the gut microbiota of mock-treated and infected young and aged mice. **(c)** Relative abundances of bacterial families in the gut microbiota of mock-treated and infected young and aged mice. Main families are represented, with all remaining families grouped under “others”. **(d)** Relative abundances (RA) of the genera *Akkermansia* and *Faecalibaculum* in the gut microbiota of mock-treated and infected young and aged mice. **(e)** Numbers of induced or repressed functional pathways identified in the comparison of mock-treated and infected (from 0 to 4 dpi, from 0 to 7 dpi, from 0 to 14 dpi and from 0 to 28 dpi) young and aged mice by PICRUSt2 analysis (enriched KEGGs). n=7 animals per group and time point, except for n=4 aged mice at 28 dpi. For **a**, **b**, and **d**: Data are expressed as mean ± SEM. Statistical analysis was performed using a two-sided Mann-Whitney test, with ^#^ indicating *P* values for age group comparisons (^#^*P* < 0.05, ^##^*P* < 0.01, ^###^*P* < 0.001) and ^*^ indicating *P* values for mock-treated vs. infected group comparisons (^*^P < 0.05, ^**^P < 0.01, ^***^P < 0.0001). *P* < 0.05 was considered statistically significant.

We then conducted a kinetic analysis of the impact of influenza on gut microbiota. Following infection, young mice showed a significant increase in gut bacterial α-diversity at 4, 7 and 28 dpi, while aged mice only exhibited this increase at 28 dpi (Fig. 5a). Phylum-level analysis revealed notable differences between age groups. In young mice, significant decreases were observed in Bacillota (formerly Firmicutes^47^) at 4, 7 and 28 dpi, and in Pseudomonadota at 4 dpi (Supplementary Fig. 6a), while Bacteroidota (formerly Bacteroidetes^47^) increased at 4 and 7 dpi, and Verrucomicrobiota increased from 4 dpi to 14 dpi, as previously reported^21,22^ (Fig. 5b). In stark contrast, aged mice showed significant shifts only in Verrucomicrobiota, with a pronounced decrease at 4 dpi followed by an increase at 28 dpi. These temporal differences between young and aged mice were even more apparent at lower taxonomic levels, specifically at the family level (Fig. 5c and Supplementary Fig. 6b). At 4 dpi, young mice exhibited significant decreases in the Oscillospiraceae, Erysipelotricaceae, and RUG350 families, alongside increases in Akkermansiaceae, Peptostreptococcaceae, Rikenellaceae, Clostridiaceae, Burkholderiaceae, and Bacteroidaceae. In contrast, aged mice showed a slight trend toward reduced Akkermansiaceae (P=0.0571) and a significant decrease in Muribaculaceae, with notable increases in Clostridiaceae, Ruminococcaceae, and Bacteroidaceae. At 7 dpi, young mice maintained most changes observed at 4 dpi, whereas aged mice exhibited only a significant decrease in the UBA932 family. By 28 dpi, young mice retained the changes seen at 4 and 7 dpi, indicating prolonged infection-driven alterations in gut microbiota composition. In contrast, at 28 dpi, aged mice showed significant increases in Oscillospiraceae and Clostridiaceae and a significant decrease in Erysipelotricaceae, changes not observed at 4 dpi. At the genus level (Table 3, Table 4, Fig. 5d, and Supplementary Fig. 6c), young mice showed significant increases in *Akkermansia*, *Romboutsia*, *Alistipes*, and *Clostridium*, along with decreases in *Lawsonibacter*, *Faecalibaculum*, *Acetatifactor*, *Kineothrix*, and *Dubosiella* at 4 and 7 dpi. Aged mice similarly showed decreased *Acetatifactor* and *Dubosiella*, and increased *Clostridium* (Supplementary Fig. 6c). Notably, aged mice exhibited an early decrease in *Akkermansia* and increase in *Faecalibaculum* (Fig. 5d), contrasting with younger mice, alongside increased levels of *Lacrimispora* and *Muribaculum*, which were not observed in young mice (Table 3 and Table 4). We then investigated the functional implications of these infection-induced changes in gut microbial composition in young and aged mice. Using PICRUSt2, we inferred the potential functions of bacterial communities in infected mice at 4, 7, 14 and 28 dpi, compared to mock-treated controls (Supplemental File 1). In aged mice, only a few metabolic pathways were identified: 1 enriched and 2 depleted at 4 dpi, and 1 enriched and 1 depleted at 7 dpi. In contrast, young mice exhibited numerous altered (mostly increased) metabolic pathways at various post-infection time points: from 0–4 dpi, 31 pathways enriched and 11 depleted; from 0–7 dpi, 38 pathways enriched and 9 depleted; from 0–14 dpi, 3 pathways depleted; and from 0–28 dpi, 26 pathways enriched and 6 depleted (Fig. 5e). Venn diagrams illustrate the increased and decreased pathways (Supplementary Table 5 and Supplementary Table 6, respectively) at different post-infection time points in young mice (Supplementary Fig. 6d). Based on KEGG pathway analysis, during early infection (4 and 7 dpi), xenobiotic degradation and metabolism pathways (i.e., caprolactam and fluorobenzoate degradation) were decreased in the gut microbiota of aged mice. In contrast, in young mice, metabolism-related pathways (including fatty acid metabolism, the TCA cycle, and the adipocytokine signaling pathway) were enriched, while the steroid biosynthesis pathway was decreased (Supplementary Fig. 7a, and Supplemental File 1). Interestingly, the adipocytokine signaling pathway exhibited opposite changes upon infection between young and aged mice, particularly at 4 and 7 dpi (Supplementary Fig. 7b), illustrating the age-dependent impact of infection on the abundance of gut bacteria that produce metabolites involved in WAT function, such as amino acids, short-chain fatty acids (SCFAs) and bile acids^45^.

**Table 3:** Impact of influenza infection on gut microbial genera in young mice.

**Genus (%)**	**Mock**	**4 dpi**	**7 dpi**	**14 dpi**	**28 dpi**
*Lawsonibacter*	15.43 ± 5.83	8.46^*^ ± 4.02	9.87^*^ ± 3.01	10.54 ± 6.79	13.04 ± 2.80
*Akkermansia*	0.004 ± 0.01	13.88^***^ ± 9.93	4.16^***^ ± 4.54	13.83^***^ ± 15.84	9.80^*^ ± 12.02
*Acetatifactor*	8.41 ± 2.55	4.41^*^ ± 3.03	5.72 ± 2.59	7.10 ± 3.46	8.07 ± 3.02
*Faecalibaculum*	13.15 ± 7.65	4.97^*^ ± 4.58	11.27 ± 10.82	7.22 ± 4.23	1.82^***^ ± 1.99
*Romboutsia*	0.39 ± 0.62	3.99^**^ ± 2.36	6.52^**^ ± 8.49	1.88 ± 2.00	7.41^***^ ± 2.92
*Alistipes*	5.76 ± 0.98	11.94^**^ ± 6.07	10.01^*^ ± 3.65	8.08 ± 5.04	10.69^**^ ± 3.41
*Kineothrix*	8.72 ± 9.41	1.87^*^ ± 2.09	2.78 ± 1.78	1.82^*^ ± 2	2.07 ± 1.37
14-2	1.63 ± 0.68	4.90^*^ ± 2.91	4.31^**^ ± 2.27	1.20 ± 0.85	1.78 ± 1.06
UMGS1872	9.52 ± 2.65	4.48^**^ ± 1.31	4.31^*^ ± 3.49	4.49^*^ ± 3.14	7.24 ± 2.83
*Anaerosacchariphilus*	2.16 ± 0.66	4.50 ± 2.85	2.58 ± 1.43	2.80 ± 1.83	4.61 ± 3.04
*Clostridium*	0.33 ± 0.41	2.51^*^ ± 2.26	4.84^***^ ± 3.31	0.43 ± 0.34	3.72^**^ ± 3.66
UBA7173	1.66 ± 1.01	5.39^**^ ± 2.77	4.68^**^ ± 1.86	3.41 ± 1.97	2.10 ± 1.40
*Lacrimispora*	2.33 ± 1.64	3.26 ± 1.97	2.96 ± 2.65	2.48 ± 1.95	3.01 ± 1.70
*Anaerotignum*	1.03 ± 0.38	0.97 ± 0.31	0.99 ± 0.40	1.16 ± 0.78	0.92 ± 0.31
RC9	1.25 ± 0.98	1.67 ± 1.20	1.67 ± 1.21	5.82 ± 6.10	0.57 ± 0.66
*Odoribacter*	3.67 ± 1.84	3.56 ± 1.72	3.35 ± 1.68	0.93^*^ ± 1.15	3.05 ± 1.28
*Sutterella*	2.46 ± 1.17	1.04^*^ ± 0.51	1.56 ± 1.60	1.83 ± 1.92	2.49 ± 1.66
*Dubosiella*	2.05 ± 1.56	0.93 ± 0.93	0.62^*^ ± 0.32	1.83 ± 2.30	1.62 ± 1.47
*Muribaculum*	1.28 ± 0.45	1.01 ± 0.56	1.52 ± 0.95	1.06 ± 1.25	0.80 ± 0.42
UBA9502	1.19 ± 0.70	1.36 ± 0.55	1.42 ± 0.27	1.27 ± 1.66	1.99 ± 0.50
Others	14.86 ± 3.77	13.34 ± 1.57	12.21 ± 1.79	17.76 ± 3.82	11.34 ± 1.37

Genus-level bacterial relative abundances (%), expressed as mean ± SEM, in young mice caecum at 0 (Mock), 4, 7, 14 and 28 dpi, n=7 at each timepoint. Comparisons to mock were assessed via two-sided Mann-Whitney tests, with significance set at *P* < 0.05. Significant differences are indicated by superscript symbols (^*^P < 0.05, ^**^P < 0.01, ^***^P < 0.001).

**Table 4:** Impact of influenza infection on gut microbial genera in aged mice.

**Genus (%)**	**Mock**	**4 dpi**	**7 dpi**	**14 dpi**	**28 dpi**
*Lawsonibacter*	5.52^###^ ± 2.18	7.33 ± 3.24	4.55^#^ ± 3.25	4.63 ± 1.77	8.74^#^ ± 2.4
*Akkermansia*	1.01^##^ ± 0.86	0.3^###^ ± 0.4	3.45 ± 6.87	3.97 ± 4.81	3.95 ± 3.86
*Acetatifactor*	16.54^##^ ± 6.85	10.36^#^ ± 3.74	8.13^**^ ± 3.24	8.99 ± 4.93	13.61^#^ ± 2.75
*Faecalibaculum*	_0_### _±0_	0.55^#^ ± 1.18	0.72^**,##^ ± 0.68	_0_### _±0_	0^##^ ± 0
*Romboutsia*	0.89 ± 1.14	_0_### _±0_	_0_### _±0_	0.84 ± 1.11	3.56 ± 2.33
*Alistipes*	3.58^#^ ± 1.74	4.44^##^ ± 1.72	5^#^ ± 3.16	3.38 ± 1.06	4.46^#^ ± 1.72
*Kineothrix*	23.08^#^ ± 10.79	16.18^##^ ± 10.41	29.96^#^ ± 17.75	18.08^##^ ± 12.74	9.68^*,##^ ± 3.45
14-2	1.19 ± 0.75	1.07^##^ ± 0.81	1.08^##^ ± 0.77	2.31 ± 1.23	3.45 ± 2.34
UMGS1872	3.58^##^ ± 2.74	8.42^**,#^ ± 3.08	4.74 ± 2.62	8.8 ± 6.53	8.58^*^ ± 2.47
*Anaerosacchariphilus*	1.73 ± 0.6	2.24 ± 1.16	0.88^*,##^ ± 0.51	1.65 ± 0.63	1.81 ± 0.63
*Clostridium*	0.05 ± 0.1	0.91^***^ ± 0.62	0.76^##^ ± 1.31	0.75^*^ ± 1.34	0.65^**,##^ ± 0.19
UBA7173	0.41^##^ ± 0.65	0.13^###^ ± 0.23	1.44^#^ ± 1.59	1.98^*^ ± 2.08	0.5^#^ ± 0.3
*Lacrimispora*	0.9 ± 0.77	2.52^*^ ± 1.08	0.45^##^ ± 0.58	1.6 ± 1.19	3.52 ± 2.64
*Anaerotignum*	0.63 ± 0.55	0.4^#^ ± 0.32	0.76 ± 0.2	0.84 ± 0.48	0.37^#^ ± 0.28
RC9	8.26^##^ ± 5.04	9.4^##^ ± 5.37	3.17^*^ ± 2.53	14.72^*,#^ ± 7.7	7.2^#^ ± 3.7
*Odoribacter*	3.01 ± 1.27	2.96 ± 2.68	2.49 ± 1.77	1.58 ± 1.16	1.8 ± 1.06
*Sutterella*	1.06^#^ ± 0.66	1.45 ± 1.05	1.1 ± 1.06	2.29 ± 2.29	3^**^ ± 0.65
*Dubosiella*	0.79 ± 0.93	0.13 ± 0.22	0.05^*,##^ ± 0.08	0.1^*,#^ ± 0.12	0.32^#^ ± 0.48
*Muribaculum*	0.33^##^ ± 0.5	0.1^###^ ± 0.1	1.28^*^ ± 0.96	0.16 ± 0.13	0.96 ± 1.55
UBA9502	1.33 ± 0.66	1.69 ± 0.93	1.02 ± 0.75	0.88 ± 0.76	2.22^*^ ± 0.68
Others	26.1^##^ ± 6.4	29.42^###^ ± 6.06	28.84^##^ ± 19.01	22.4 ± 4.44	21.61^##^ ± 1.3

Genus-level bacterial relative abundances (%), expressed as mean ± SEM, in aged mice caecum at 0 (Mock), 4, 7, 14 and 28 dpi, n=7 at each timepoint, except for n=4 at 28 dpi. Groups were compared using a two-sided Mann-Whitney test, with ^#^ indicating *P* values for age group comparisons (with Table 3) (^#^*P* < 0.05, ^##^*P* < 0.01, ^###^*P* < 0.001), and ^*^ indicating *P* values for mock vs. infected group comparisons (^*^P < 0.05, ^**^P < 0.01, ^***^P < 0.001). *P* < 0.05 was considered statistically significant. Significant differences are indicated by superscript symbols.

**Fig. 6:**
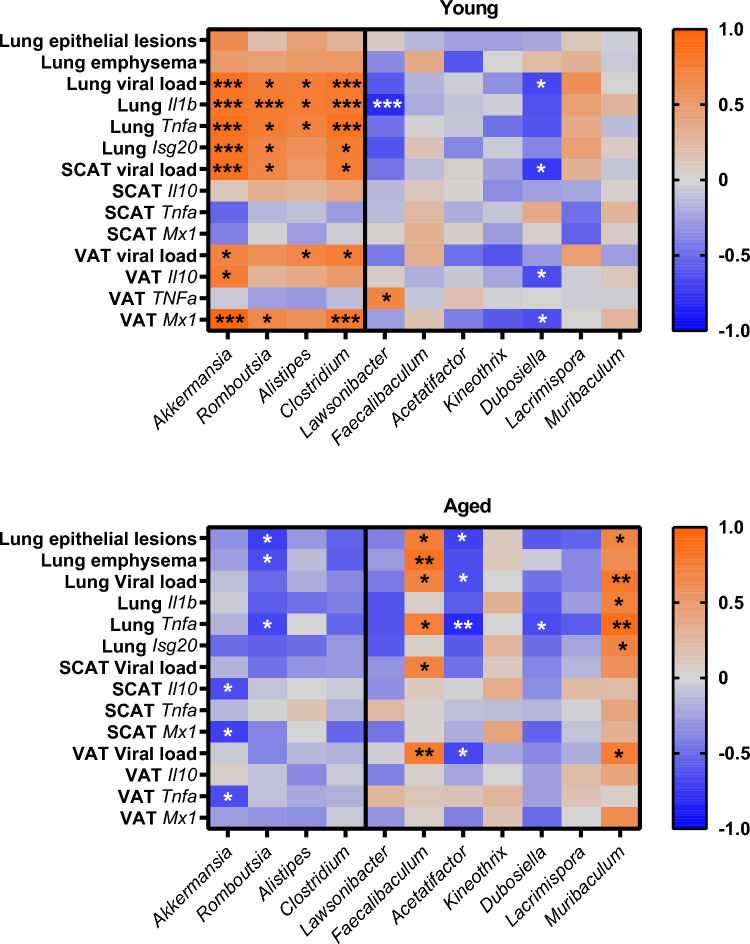
Correlations at 7 dpi between gut bacteria genera, and lung and WAT responses to infection. Heatmap representing Spearman’s correlations between relative abundances of bacterial genera, and lung and WAT features (7 dpi), in young and aged mice, n=7 animals per group. Blue and red colors stand for inverse and positive correlations, respectively. The symbols ^*^ on plots represent significant correlations (^*^P < 0.05, ^**^P < 0.01, ^***^P < 0.001). *P* < 0.05 was considered statistically significant.

Putative associations between bacterial genera and features of the lungs and WAT at 7 dpi were evaluated using Spearman correlation tests and the identification of associated blocks on a heatmap (Fig. 6). In young mice, a statistically significant block, which includes *Akkermansia*, *Romboutsia*, *Alistipes* and *Clostridium*—all of which increased upon infection in this age group—was positively correlated with lung viral load, inflammation and anti-viral response, SCAT viral load, VAT viral load, and VAT *Mx1* expression. No bacterial genera were correlated with lung pathology in this age group. In contrast, in aged mice, *Muribaculum* and *Faecalibaculum*, which increased exclusively in older mice, were positively correlated with lung pathology.

Overall, influenza infection triggers early alterations in gut bacterial composition and function that markedly differentiate young mice from aged mice. This distinction is primarily characterized by contrasting shifts in specific bacterial genera: at 4 dpi, *Akkermansia* increases only in young mice, while at 7 dpi, *Faecalibaculum* and *Muribaculum* expand exclusively in aged mice, with their abundance correlating with lung pathology at this timepoint.

### Influenza differently dysregulates the serum metabolomic profiles of young mice and aged mice

To investigate if influenza-related changes in gut microbiota, lungs, and WAT align with shifts in blood metabolite profiles, we performed a targeted metabolome analysis at 7 dpi (acute phase of infection). We analyzed serum samples from mock-treated and IAV-infected young and aged mice using liquid chromatography-tandem mass spectrometry (LC-MS/MS).

Partial least squares-discriminant analysis (PLS-DA) of serum compositions from non-infected young and aged mice distinctly separated the two age groups (Supplementary Fig. 8a), revealing age-related changes in serum metabolite profiles, despite no statistically significant differences (PERMANOVA test, *P* > 0.05). A linear model analysis identified 58 metabolites that differed significantly between young and aged mice (*P* < 0.05), including two amino acid-related compounds, trans-4-hydroxyproline (t4-OH-Pro) and cis-4-hydroxyproline (c4-OH-Pro), both of which were reduced in aged mice (adjusted *P* < 0.01) (Supplementary Fig. 8b, and Supplemental File 2). Of these 58 metabolites, 42 were down-regulated and 16 were up-regulated in aged mice. Random forest (RF) analysis pinpointed 15 metabolites that contributed most to classification accuracy (Mean Decrease Accuracy), aiding in the distinction between aged and young mice (Supplementary Fig. 8c). These findings indicate age-related alterations in the metabolism of amino acids (3-Methyl-L-Histine, β-Alanyl-3-methylhistidine (also known as anserine), and hydroxyproline), lipids (ceramides, phosphatidylethanolamines), purines (hypoxanthine, xanthine), and bile acids (taurocholic acid, tauromuricholic acid), consistent with previous reports^49,50^.

**Fig. 7:**
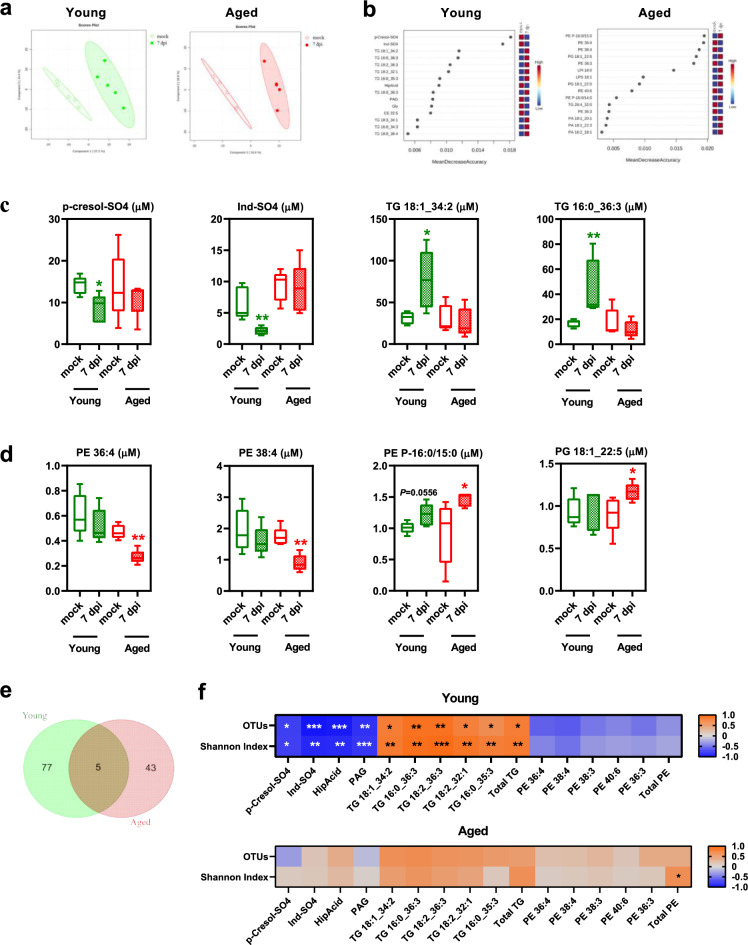
Influenza infection induces changes in the serum metabolome that differentiate young mice from aged mice. Serum samples from mock-treated and 7-dpi-infected young and aged mice were analyzed, n=5 per group. **(a)** PLS-DA scores for metabolite profiling data in young mice (PERMANOVA, *P* value = 0.019) (left), and aged mice (PERMANOVA, *P* value = 0.071) (right). **(b)** Random forest ranking of the top- 15 predictive metabolites for infection status in young (left) and aged (right) mice. **(c**, **d)** Boxplots displaying the average serum concentrations (μM) of the top-4 metabolites ranked by random forest analysis for young **(c)** and aged **(d)** mice. **(e)** Venn diagrams (https://bioinformatics.psb.ugent.be/) illustrating distinct or overlapping lipid species modulated upon infection in young and aged mice. **(f)** Heatmap representing Spearman’s correlations between gut microbial alpha diversity (OTUs and Shannon index) and blood metabolites (7 dpi), in young mice (above) and aged mice (below), n=5 per group. Blue and red colors stand for inverse and positive correlations, respectively. The symbols ^*^ on plots represent significant correlations. Groups were compared using a two-sided Mann-Whitney test, with ^*^ indicating *P* values for mock vs. infected group comparisons (^*^P < 0.05, ^**^P < 0.01, ^***^P < 0.001). *P* < 0.05 was considered statistically significant.

Influenza infection led to significant changes in serum metabolite profiles, as shown by PLS-DA loading plots, which reveal clear distinctions between infected and mock samples in both age groups. In young mice, PERMANOVA analysis confirmed statistically significant differences (*P* < 0.05). In aged mice, although the *P*-value was 0.07, the distinct separation of clusters indicates notable differences in metabolite patterns (Fig. 7a). Heatmaps of differential metabolite concentrations (*P* < 0.05, linear model) between mock-treated and infected mice showed clear changes, identifying 88 metabolites in young mice and 50 in aged mice, visualized through hierarchical clustering (One Minus Pearson Correlation, Average linkage). In young mice, 81 metabolites increased significantly and 7 decreased, whereas in aged mice, 13 metabolites increased and 37 decreased compared to respective mock-treated controls (Supplementary Fig. 9a, and Supplemental File 2). RF analyses ranked metabolites based on their predictive importance for infection status in both young and aged mice (Fig. 7b). In young mice, the highest-ranking metabolites were cresols (p-Cresol-SO4) and indole-derivatives (Ind-SO4), which decreased with infection, and glycerolipids (triglycerides), which increased (Fig. 7b, left). Remarkably, in aged mice, the discriminative metabolites were all glycerophospholipids (GPL) (Fig. 7b, right), notably phosphatidylethanolamines (PE), which decreased upon infection (Supplementary Fig. 9b). Serum concentrations of the top-4 RF-ranked metabolites confirmed an age-dependent response to infection. In young mice, infection led to reduced levels of p-Cresol-SO4 and Ind-SO4, and increased levels of TG 18:1_34:2 and TG 16:0_36:3 (Fig. 7c). In contrast, aged mice exhibited decreased levels of PE 36:4 and PE 38:4, with increased levels of PE P-16:0/15:0 and PG 18:1_22:5 in response to infection (Fig. 7d). Venn diagrams comparing significantly altered lipids in infected young and aged mice revealed both unique and shared responses (Fig. 7e, and Supplementary Table 7). Lipid Pathway Enrichment Analysis highlighted GPL metabolism as significantly associated with altered lipids in infected aged mice (Table 5).

**Table 5:** Enrichment analysis results for lipids associated with influenza infection.

**Pathway name**	**Pathway lipids**	***P*-value**	**Benjamini** **correction**	**Bonferroni** **correction**
**Only in young mice**				
Steroid biosynthesis	41	0,475212914	0,475212914	1
Glycosylphosphatidylinositol (GPI)- anchor biosynthesis	3	0,044437899	0,255829686	0,62213058
Inositol phosphate metabolism	9	0,128159538	0,256319077	1
Ether lipid metabolism	16	0,21766311	0,304728354	1
Autophagy - animal	4	0,058861477	0,255829686	0,824060685
Autophagy - other	3	0,044437899	0,255829686	0,62213058
Glycerophospholipid metabolism	26	0,331556604	0,357060958	1
Phosphatidylinositol signaling system	11	0,154602469	0,27055432	1
Fat digestion and absorption	8	0,114668777	0,256319077	1
Ovarian steroidogenesis	18	0,241710471	0,307631508	1
Cholesterol metabolism	8	0,114668777	0,256319077	1
Vitamin digestion and absorption	15	0,205391159	0,304728354	1
Tuberculosis	5	0,073094196	0,255829686	1
Bile secretion	25	0,320861509	0,357060958	1
**Both in young and aged mice**				
**Glycerophospholipid metabolism**	**26**	**0,048780488**	**0,048780488**	**0,048780488**
**Only in aged mice**				
Linoleic acid metabolism	25	0,584873217	0,63804351	1
Steroid biosynthesis	41	0,768979711	0,802413611	1
Ether lipid metabolism	16	0,427475712	0,603495123	1
Glycosylphosphatidylinositol (GPI)- anchor biosynthesis	3	0,003172422	0,025379377	0,07613813
Sphingolipid metabolism	21	0,520775393	0,63804351	1
Inositol phosphate metabolism	9	0,267708969	0,458929661	1
**Glycerophospholipid metabolism**	**26**	**6,29558E-06**	**0,000151094**	**0,000151094**
alpha-Linolenic acid metabolism	23	0,553910381	0,63804351	1
Arachidonic acid metabolism	75	0,937830501	0,937830501	1
Sphingolipid signaling pathway	9	0,267708969	0,458929661	1
Autophagy - other	3	0,003172422	0,025379377	0,07613813
Autophagy - animal	4	0,006217538	0,029844181	0,149220907
Phosphatidylinositol signaling system	11	0,317201381	0,50752221	1
Necroptosis	4	0,128738663	0,380123056	1
Ferroptosis	11	0,004519	0,027114	0,108456001
Retrograde endocannabinoid signaling	8	0,026762258	0,107049032	0,64229419
Ovarian steroidogenesis	18	0,466685294	0,622247059	1
Fat digestion and absorption	8	0,241710471	0,458929661	1
Vitamin digestion and absorption	15	0,406864838	0,603495123	1
Cholesterol metabolism	8	0,241710471	0,458929661	1
Tuberculosis	5	0,158384607	0,380123056	1
Kaposi’s sarcoma-associated				
herpesvirus infection	3	0,098108382	0,336371595	1
Choline metabolism in cancer	5	0,158384607	0,380123056	1
Bile secretion	25	0,584873217	0,63804351	1

Lipid pathway enrichment analysis (LIPEA) on the lipids that are modulated upon influenza infection in young mice only (77 lipids), in both young and aged mice (5 lipids), and in aged mice only (43 lipids). Grey shading indicates *P* < 0.05, and bolded and shaded values denote *P* and adjusted *P* values (Benjamini and Bonferroni corrections) < 0.05.

Using pairwise Spearman correlation analyses, we investigated the relationships between gut bacterial richness and discriminating blood metabolites in young and aged mice at 7 dpi (Fig. 7f). In young mice, characterized by higher microbial diversity upon infection, we observed positive correlations between α-diversity and TG species as well as with total TG levels. Conversely, p-Cresol-SO4, Ind-SO4, hippuric acid (HipAcid), and phenylacetylglutamine (PAG) correlated negatively with bacterial richness. In aged mice, who exhibit lower microbial diversity upon infection, only total PE levels showed a positive correlation with bacterial richness.

These results reveal age-specific metabolic responses to influenza in mice. In young mice, infection significantly reduces circulating levels of p-Cresol-SO4, Ind-SO4, HipAcid, and PAG, while increasing triglyceride levels. In contrast, aged mice show disrupted GPL metabolism, with a particular impact on PEs. These findings highlight age-dependent metabolic alterations in response to influenza and identify PE species as metabolites of potential relevance during infection.

### Age-dependent correlations between blood metabolites and lung, WAT, and bacterial genera during influenza

**Fig. 8:**
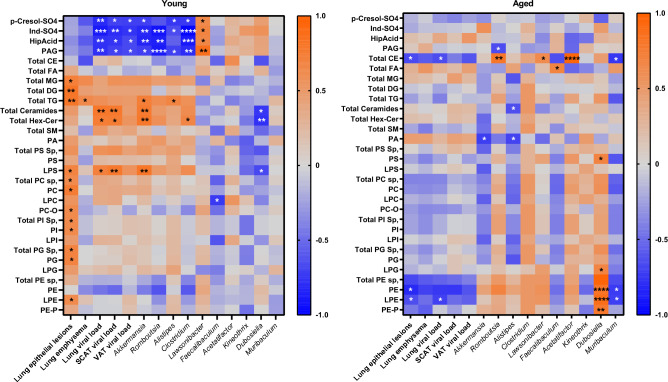
Correlations at 7 dpi between serum metabolites, and gut bacteria genera, lung pathology and viral loads. Heatmap representing Spearman’s correlations between relative abundances of bacterial genera, lung pathology, lung, SCAT and VAT viral loads, and blood metabolites (7 dpi), in young and aged mice, n=5 per group. Blue and red colors stand for inverse and positive correlations, respectively. The symbols^*^ on plots represent significant correlations (^*^P < 0.05, ^**^P < 0.01, ^***^P < 0.001, ^****^P < 0.0001). *P* < 0.05 was considered statistically significant.

To explore the relationships between blood metabolites and lung, SCAT, and VAT features as well as with bacterial genera discriminating the two age groups during infection, we performed pairwise Spearman correlation analyses (Figure 8). In young mice, p-Cresol-SO4, Ind-SO4, HipAcid, and PAG showed negative correlations with tissue viral load, *Akkermansia*, *Romboutsia*, *Alistipes* and *Clostridium* abundances. Conversely, these metabolites showed positive correlations with *Lawsonibacter.* Notably, lung epithelial lesions showed positive correlations with various lipid classes, except PE, in this age group. In striking contrast, lung epithelial lesions correlated negatively with PE and lysophosphatidylethanolamine (LPE) species in aged mice. Noteworthily, PE and LPE levels showed positive and negative correlations with, respectively, *Dubosiella* and *Muribaculum*, both of which increased upon infection exclusively in aged mice.

## Discussion

In this study, we investigated the impact of aging on the gut-lung-adipose tissue axis’s response to experimental influenza in mice. Our findings reveal significant age-related differences in the response to infection and identify factors and pathways associated with greater disease severity in aged mice.

At steady state (before infection), aged mice had lower gut microbiota diversity than younger mice, aligning with previous studies^15,51,52^. A diverse gut microbiota is a key indicator of health and resilience^53^, while age-related microbial alterations—characterized by loss of diversity and/or changes in composition, commonly referred to as dysbiosis—have been linked to a higher risk of infections^54,55^. The impact of pre-existing age-related gut dysbiosis on influenza remains unclear. However, aging is associated with a decline in microbial-derived metabolites that were reported to protect against respiratory infections, such as SCFAs^21,56^, indole-3-propionate acid^22^, desaminotyrosine^57^, and secondary bile acids (BAs)^58^. This decline may contribute to increased disease severity in older adults. Following infection, young mice exhibited a rapid (4 dpi) and sustained (28 dpi) increase in gut microbiota diversity, whereas aged mice showed minimal changes. Notably, the genus *Akkermansia*, primarily represented by *Akkermansia muciniphila*^59^, responded differently to infection across age groups. In young mice, *Akkermansia* levels increased, consistent with our previous findings^21,22^, while in aged mice no such increase was observed—a previously undocumented phenomenon. The rise in *Akkermansia* in young mice during infection is particularly notable due to its established benefits, such as strengthening the gut mucosal barrier, regulating metabolism, and reducing inflammation^60,61^. Consistently, supplementation with *A. muciniphila* has been shown to improve outcomes in influenza-infected young mice, suggesting its protective role during infection^62^. Thus, the early rise in *Akkermansia* in young mice may be part of an adaptive response of the gut microbiota to infection, while its absence in aged mice could indicate an impaired or maladaptive response. Furthermore, this finding supports the potential of *A. muciniphila* as a probiotic therapy^63^ to boost immune function and improve resistance to influenza, particularly in older individuals.

Given that the gut microbiota and its metabolites, including SCFAs and secondary BAs, regulate WAT’s physiological function via the gut-adipose tissue axis^24,64–67^, we investigated the impact of infection on SCAT and VAT depots. In young mice, infection transiently triggered SCAT browning—a response not seen in aged mice, indicating a reduced capacity for WAT browning in older individuals^68,69^. This deficiency may partly stem from age-related declines in SCFAs^70,71^ and secondary BAs^72^, which are known to support WAT browning^67,73,74^. In VAT, young mice displayed a late increase in adipocyte size, while aged mice exhibited a reduction. These findings indicate distinct metabolic responses to infection in WAT, with young mice adapting through beneficial metabolic changes, while aged mice exhibit a maladaptive metabolic response. Furthermore, the innate immune response in aged WAT (mainly VAT) was compromised, as evidenced by the reduced recruitment of NK and NKT cells compared to young mice. This impairment may potentially worsen influenza severity, as these cells are essential for controlling viral replication^75^ and modulating the CD8^+^ T cell response^76,77^. Interestingly, immune cell populations in the lungs mirrored those in VAT during both aging and infection, suggesting a potential interplay between these tissues, as proposed in previous studies^39^. To deepen our understanding, a detailed characterization of T cells in WAT is necessary to elucidate their role in influenza severity. Additionally, further research is required to clarify how elevated baseline NKT cell levels and/or reduced NK cell levels in aged VAT contribute to increased susceptibility to severe influenza, a pattern also observed in other tissues and blood^78,79^.

Strikingly, infection triggered distinct blood metabolite shifts in young and aged mice at 7 dpi, some of which correlated with changes observed in the lungs, gut microbiota, and WAT at the same timepoint. In young mice, infection led to a decrease in p-Cresol-SO4 and Ind-SO4, suggesting a decline in p-Cresol- and indole- producing bacterial families like *Clostridiaceae* and *Enterobacteriaceae* in the gut microbiota^80^. Given the harmful effects of these uremic toxins, such as promoting inflammation, endothelial dysfunction, and oxidative stress^81^, this reduction in young hosts likely provide a benefit in coping with infection. Notably, p-Cresol-SO4 and Ind-SO4 levels negatively correlated with bacterial diversity in young mice. Besides, infection led to an increase in blood triglyceride levels in young mice, whereas aged mice showed altered glycerophospholipids (GPL) levels, particularly phosphoethanolamines (PEs), which are associated with inflammation^82^ and diseases, including respiratory viral infections^83,84^. In line with our findings, Ohno and colleagues, in their analysis of blood lipidomic profiles across various doses of IAV, noted an increase in specific PEs, particularly polyunsaturated-fatty-acid-containing PEs, during lethal infection^85^. These changes in lipid profiles in aged mice suggest that lipid metabolism might contribute to the pathophysiology of severe influenza, potentially paving the way for more tailored therapeutic interventions.

Our study presents a novel, integrative approach that reveals associations between WAT, the blood metabolome, and gut microbiota changes in relation to influenza outcomes in young and aged mice. Our findings suggest that severe influenza in aged mice may be driven by age-specific disruptions in lung inflammation, gut microbiota diversity and composition, and WAT metabolic and immune functions. Compared to young mice, aged mice failed to exhibit SCAT browning and displayed increased inflammation along with impaired innate responses in VAT. Moreover, unlike their younger counterparts, aged mice did not experience an increase in gut bacterial diversity or *Akkermansia* abundance. Instead, they showed a distinct rise in *Faecalibaculum* and *Muribaculum*, which was positively correlated with lung damage at 7 dpi (but not at 14 dpi, data not shown). However, correlation does not imply causality, and further mechanistic studies are needed to establish whether *Facealibaculum* and/or *Muribaculum* play a direct role in mediating or responding to lung pathology in aged mice. Strikingly, these infection-induced changes in the lungs, WAT, and gut microbiota coincided with a distinct blood GPL profile in aged mice, suggesting potential cross-system interactions that may contribute to increased influenza severity in the elderly. Our study has limitations. Although our findings suggest potential links between age-related gut dysbiosis (e.g., reduced diversity, *A. muciniphila* abundance), WAT features (e.g., SCAT browning, reduced innate immunity), blood metabolites (e.g., p-Cresol-SO4, Ind-SO4, lipids), and influenza outcomes, causality remains to be established. Various experimental models, including germ-free or antibiotic-treated mice, gnotobiotic systems, fecal microbiota transplantation, and microbial metabolite supplementation, have been used to investigate the causal role of the microbiome on influenza infection^57,86,87^ and in shaping metabolite profiles, such as lipids species including PEs^88^. Future studies in aged mice could explore whether (i) consuming specific dietary components like fibers and prebiotics, which enhance gut microbial diversity^89^, (ii) oral supplementation with *Akkermansia muciniphila*, known to protect against influenza in young mice^62^, or (iii) various conditions/agents that promote WAT browning, including physical activity^90^, treatment with retinoic acid^91^, or dietary supplementation with flavonoids^92^ or marine-derived n-3 polyunsaturated fatty acids^93^, can improve disease severity. Another limitation is that our metabolomic analysis did not include SCFAs, which are known to decline with aging^70,71^, due to plasma volume constraints. Given their role in reducing inflammation and acute lung injury in aged mice^94^, and their reported depletion during influenza^21^, SCFA levels likely inversely correlate with influenza severity. Although our study was conducted in a mouse model, the findings provide insights that may be relevant to human aging and influenza infection. In older adults, the gut microbiota tends to be less diverse, with a higher abundance of pro-inflammatory species and fewer beneficial bacteria^95^. While human studies specifically investigating age-related changes in the gut microbiota in the context of influenza are still limited, some evidence links microbiota composition to response to vaccination^96^, and several clinical trials have explored the effects of pro- and pre-biotics on influenza vaccine outcomes^97^. Additionally, aged-related shifts in the blood metabolome have been observed in infected individuals^98^, and changes in WAT, such as those observed in individuals with obesity, can worsen influenza severity^99^. Taken together, our findings highlight the therapeutic potential of modulating the gut microbiota in the elderly to enhance its diversity and selectively increase the abundance of *Akkermansia*. They also suggest the possibility of targeting WAT’s metabolic and immune functions and provide new insights into the role of GPLs in influencing disease severity.

## Methods

### Ethics statement

Animal studies were performed in accordance with the ARRIVE guidelines recommendations and the care, use, and treatment of mice were in agreement with current national and institutional regulations and ethical guidelines (Animal Experimentation and High Technology Platform of the *Institut Pasteur de Lille*, Agreement # E59-350009). All mouse procedures were approved by the institutional regional ethics committee “*Comité d’Ethique en Experimentation Animale*” (CEEA)75. Animal studies were authorized by the French Ministry of Education, Research and Innovation (protocol APAFIS#: 2020013113414570_v2).

### Animals, virus, and infection protocol

Young-adult (2-month-old, designated as “young”) and aged (18-month-old) C57BL/6JRj male mice were purchased from Janvier Labs (Le Genest-Saint-Isle, France) and housed in an Animal Biosafety Level-2 facility under controlled conditions (12:12 dark-light cycle, 18–22°C temperature, 55% ± 10 humidity of 55%) and with enriched environment. Mice had *ad libitum* access to rodent chow (D12450B, with 10% kcal% fat, Research Diets, New Brunswick, NJ, USA) and water. The human-derived, mouse-adapted influenza A/Scotland/20/1974 (H3N2) was grown, isolated, and titrated in Madin-Darby Canine Kidney (MDCK) cells. For infection, mice were anesthetized with ketamine (1.3 mg) (Imalgene^®^1000, Boehringer Ingelheim Animal Health France SCS, Lyon, France) and xylazine (0.26 mg) (Rompun^®^ 2%, Elanco GmbH, Cuxhaven, Germany) in phosphate buffered saline (PBS) (100 μL) via intraperitoneal injection, then intranasally inoculated with either a sublethal dose (50 Plaque Forming Units (PFUs) diluted in 50 μL PBS) of H3N2 (infected groups) or PBS (mock-treated groups). The study design is presented Supplementary Fig. 1a: Two independent infection experiments were performed. In the first experiment, 42 mice per age group were used, with 35 mice per group infected. Body weight was monitored daily from 0 to 28 days post-infection (dpi). At 0 (mock-treated controls), 2, 4, 7, 14, and 28 dpi, mice were sacrificed for sample collection. The second experiment focused on flow cytometry analysis of immune cell populations in lungs, SCAT, and VAT. A total of 35 mice per age group were included, with 28 mice per group infected. At 0 (mock-treated controls), 4, 7, 14, and 28 dpi, mice per group were sacrificed for tissue collection, and preparation of cell suspensions for flow cytometry analysis.

### Sample collection

In the infection experiment 1 (Supplementary Fig. 1a), mice were monitored daily for 28 days post-infection (dpi) for signs of morbidity and changes in body weight. At specified sacrifice days (0 (mock-treated controls), 2, 4, 7, 14, and 28 dpi), mice were euthanized with an intraperitoneal injection of euthasol (40 mg/kg), and blood, lungs, adipose tissues (subcutaneous (inguinal) adipose tissue (SCAT) and visceral (epididymal) adipose tissue (VAT)), and caecal contents were collected. Lung and adipose tissue samples were either snap-frozen for gene expression, embedded in paraffin for histology and histomorphometry. Sera were stored at −80°C for cytokine and metabolomic analyses. Caecal contents were snap-frozen and stored at −80°C for microbiota analyses using 16S rRNA sequencing method.

### RNA extraction and real-time quantitative PCR

Tissue samples were homogenized using an Ultra-Turrax homogenizer (IKA-Werke GmbH & Co. KG, Staufen, Germany) in either RA1 buffer (1mL) (NucleoSpin RNA kit, Macherey-Nagel, Düren, Germany) supplemented with 1% β-mercaptoethanol for lung samples, or QIAzol lysis reagent (1 mL per 100 mg) (QIAGEN, Hilden, Germany) for adipose tissue samples. Total RNAs were then extracted using the NucleoSpin RNA kit (Macherey-Nagel), and their quantity and quality were assessed with a NanoDrop^TM^ spectrophotometer (ThermoFisher Scientific, Waltham, MA, USA) (260/280 nm, 260/230 nm). Viral RNA quantification was done using a negative strand specific RT-qPCR targeting the IAV RNA *M1* gene^34^. Briefly, total RNAs were reverse-transcribed using a primer specific for *M1* (5’-TCT AAC CGA GGT CGA AAC GTA-3’), and PCR was performed with TaqMan technology (TaqMan Universal PCR Master Mix, Applied Biosystems, Foster City, CA, USA), detection primers pairs for M1 (sense: 5’-CAA AGC GTC TAC GCT GCA GTC C-3’; anti-sense: 5’-CAA AGC GTC TAC GCT GCA GTC C-3’) and M1 specific TaqMan probe ((FAM) 5’-TTT GTG TTC ACG CTC ACC GTG CC-3’ (TAMRA)), using the QuantStudio^TM^ 5 and QuantStudio^TM^ 12 Flex Real-Time PCR Systems (Applied Biosystems).

For gene expression analysis, RT was performed using the High-capacity RNA-to-cDNA kit (Applied Biosystems), followed by PCR with Power SYBR Green PCR Master Mix (Applied Biosystems), using the QuantStudio ^TM^ 5 and QuantStudio^TM^ 12 Flex Real-Time PCR System (Applied Biosystems). For every reaction the qPCR cycling was done with a 95°C-15 seconds denaturation step and 40 cycles of 60°C-1 minute annealing/elongation, followed by a melting curve. Gene-specific primers were designed using the Primer Express^TM^ v3 software (ThermoFisher Scientific), and primer sequences are listed in Supplementary Table 8. Reactions were run in duplicate and the geometric mean of the housekeeping gene *Gapdh* (coding for glyceraldehyde-3-phosphate dehydrogenase) (for lung samples^100^) or *Eef2* (coding for eukaryotic translation elongation factor 2) (for adipose tissue samples^101^) transcript levels was used as internal control to normalize the variability in expression levels. Relative mRNA levels were quantified using the 2^−ΔΔCt^ method^102^, which involves first calculating the difference in PCR cycle threshold (Ct) values between the target and housekeeping genes (ΔCt), and then comparing these ΔCt values between infected and non-infected groups (ΔΔCt). Gene expression was normalized to housekeeping* g*ene levels and presented as fold change relative to the average expression in mock-treated young mice.

### Preparation of single cell suspensions from lungs and adipose tissues, and flow cytometry analysis

In the infection experiment 2 (Supplementary Fig. 1a), mice were sacrificed at 0 (mock-treated controls), 4, 7, 14, and 28 dpi, and the whole lungs, SCAT and VAT were harvested. Freshly collected lungs were diced into ~ 1-mm^3^ fragments and enzymatically digested for 30 minutes in DMEM supplemented with collagenase type VIII (1 mg/mL) (Sigma-Aldrich, Saint-Louis, MI, USA) at 37°C. Cold-stop DMEM was added and enzymatically digested lung tissue pieces were mechanically disrupted through a 18G needle before being centrifuged at 400 g for 7 minutes at 4°C. Cell pellets were resuspended in a 30% Percoll solution (one phase) (Percoll^TM^ GE Healthcare C, Chicago, IL, USA), and centrifuged at 500 g for 15 minutes at 4°C. Cell pellets were resuspended in red blood cell lysis buffer (Red Blood Cell Lysing Buffer Hybri-Max^TM^, Sigma-Aldrich) for 5 minutes on ice. After centrifugation at 400 g for 7 minutes at 4 °C, lung single cell suspensions were resuspended in ice-cold FACS buffer (i.e., PBS supplemented with 0.5% bovine serum albumin (BSA)) for flow cytometry staining.

For the isolation of stromal vascular fraction (SVF) from adipose tissues, freshly collected SCAT and VAT samples were minced and digested for 1 hour in DMEM supplemented with collagenase IV (1 mg/mL) (Sigma-Aldrich), under gentle agitation at 37°C. To eliminate undigested fragments and mature, lipid-filled adipocytes, digested tissues were then passed through a 280 µm nylon filter and centrifuged at 400 g for 7 minutes at room temperature. Cell pellets, which contain stromal vascular cells, were resuspended in cold red blood cell lysis buffer for 3 minutes on ice, and filtered through a 90 µm nylon filter before being centrifuged at 400 g for 5 minutes at room temperature. SVF cell suspensions were resuspended in ice-cold FACS buffer for flow cytometry staining.

Subsequently, both lung and SVF cell suspensions underwent flow cytometry analysis using a LSRFortessa^TM^ cell analyzer (BD Biosciences, Franklin Lakes, NJ, USA) using the FACSDiva^TM^ software (gating strategy is presented in Supplementary Fig. 10). Cells were first incubated with a viability marker (1/1000) (Zombie Red^TM^ Fixable Viability Kit, BioLegend, San Diego, CA, USA) for 5 minutes at room temperature before incubation with an Fc blocking reagent (1/200) (anti-mouse CD16/CD32, TruStain FcX^TM^ PLUS, BioLegend). Afterwards, cells were stained with the appropriate panel of antibodies for 30 minutes in FACS buffer at 4°C. Conjugated antibodies were used against mouse NK1.1 (PE, clone PK136, 1/400), I-A/I-E (AF700, clone M5/114.15.2, 1/600), Ly6G (APC, clone 1A8, 1/600), CD45 (BV510, clone 30-F11, 1/400), CD19 (BV605, clone 6D5, 1/400), F4/80 (FITC, clone BM8, 1/600), CD8a (PE/Cy5, clone 53-6.7.7, 1/800), CD11c (PE/Cy7, clone N418, 1/400), CD3e (PerCP, clone 145-2C11, 1/100), CD11b (BV711, clone M1/70, 1/400), CD206 (BV650, clone C068C2, 1/400) from BioLegend, and CD4 (APC-H7, clone GK1.5, 1/400) and Siglec-F (BV786, clone E50-2440, 1/400) from BD Biosciences. The phenotype to identify each cell population was as follows: Alveolar macrophages (for lungs): CD45^+^/Live-Dead^low^/Siglec-F^+^/CD11b^low^, Neutrophils: Live-Dead^-^/CD45^+^/CD11b^+^/Ly6G+*,* macrophages (interstitial macrophages for lungs): Live-Dead^-^/CD45^+^/CD11b^+^/F4/80^+^*,* M0-like macrophages (interstitial macrophages for lungs): Live-Dead^-^/CD45^+^/CD11b^+^/F4/80^+^/CD206^-^/CD11c^-^*,* M1-like macrophages (interstitial macrophages for lungs): Live-Dead^-^/CD45^+^/CD11b^+^/F4/80^+^/CD206^-^/CD11c^+^*,* M2-like macrophages (interstitial macrophages for lungs): Live-Dead^-^/CD45^+^/CD11b^+^/F4/80^+^/CD206^+^/CD11c^-^*,* Dendritic cells: Live-Dead^-^/CD45^+^/Ly6G^-^/F4/80^-^/MHC-II^hi^/CD11c^hi^*,* NK cells: Live-Dead^-^/CD45^+^/Ly6G^-^/F4/80^-^/NK1.1^+^/CD3e^-^*,* NKT cells: Live-Dead^-^/CD45^+^/Ly6G^-^/F4/80^-^/NK1.1^+^/CD3e^+^*,* T cells: Live-Dead^-^/CD45^+^/Ly6G^-^/F4/80^-^/NK1.1^-^/CD3e^+^*,* CD8^+^ T cells : Live-Dead^-^/CD45^+^/Ly6G^-^/F4/80^-^/NK1.1^-^/CD3e^+^/CD8^+^/CD4^-^*,* CD4^+^ T cell (for lungs): Live-Dead^-^/CD45^+^/Ly6G^-^/F4/80^-^/NK1.1^-^/CD3e^+^/CD8^-^/CD4^+^*,* CD8^-^ T cells (for AT): Live-Dead^-^/CD45^+^/Ly6G^-^/F4/80^-^/NK1.1^-^/CD3e^+^/CD8^-^, B cells : Live-Dead^-^/CD45^+^/Ly6G^-^/F4/80^-^/NK1.1^-^/CD3e^-^/MHC II^+^/CD19^+^ (see gating strategies Supplementary Fig. 4a (lungs), and Supplementary Fig. 10 (WAT)). Data were analysed using FlowJo^TM^ software v10.8.1 (Ashland, OR, USA).

### Lung and adipose tissue histology, and adipose tissue histomorphometry

Paraformaldehyde-fixed paraffin-embedded lung, SCAT, and VAT sections (5µm-thick) were prepared, de-paraffinized, rehydrated, and stained with hematoxylin and eosin (H&E) for histological examination. Lung histopathological scoring was performed through evaluating, on a semi-quantitative scale of 0 to 3 and in a blind manner, the degree of peribronchial and perivascular inflammation (0:, absence, 1: < 10 cells per field, 2: 10 to 40 cells per field, 3: ≥ 40 cells per field), the intensity of bronchial and vascular lesions (0: absence, 1 < 20% lesions, 2: 20 to 40% lesions, 3 ≥ 40% lesions), and the presence of edema and fibrin around vessels (0: absence, 1: < twice the size of intact blood vessel, 2: < thrice the size of intact blood vessel, 3: ≥ more than thrice the size of intact blood vessel). Alveolar inflammatory infiltrates (0: absence, 1: few cells by alveolus, 2: regular presence of inflammatory cells in each alveolus, 3: obstruction of the alveoli by inflammatory cells), alveolar wall thickening (0: < one cell thick, 1: 1 to 2 cell thick, 2: 2 to 3 cell thick, 3: ≥ 3 cell thick), and emphysematous-like lesions (0: < 10% of the alveoli, 1: < 10 to 20% of the alveoli, 2: 20 to 40% of the alveoli, 3: ≥ 40% of the alveoli) were also quantified. Each scoring was performed on 3 different fields per animal that include the different lung compartments. The total pathological score was then defined as the sum of the peribronchial (inflammation and lesion) scores, the perivascular (inflammation and lesion) scores, and the alveolar (inflammation, percentage of altered zones and alveolar wall thickening) scores.

For SCAT and VAT adipocyte size measurement by histomorphometry, at least two fields per H&E-stained slide were visualized and images were digitally captured using a digital camera (Flexacam C1 Camera, Leica Microsystems, Wetzlar, Germany) connected to an optical microscope (Leica DM3000 Led, Leica Microsystems) at 5x magnification. Adipocyte areas (in µm^2^) were averaged to determine mean cross-sectional area and binned by area to assess the distribution of cells sizes within sections (young day 0: 3 (SCAT) - 10 (VAT) animals, young day 7: 3 (SCAT) - 6 (VAT) animals, young day 28: 3 (SCAT) - 6 (VAT) animals, aged day 0: 3 (SCAT) - 8 (VAT) animals, aged day 7: 3 (SCAT) - 6 (VAT) animals, and aged day 28: 3 (SCAT and VAT) animals. An average of 2213 (for SCAT) or 1212 (for VAT) adipocytes per tissue sample was measured with a range of 1261-3192 (for SCAT) or 596–1558 (for VAT) adipocytes per tissue sample. Adipocytes with disrupted or incomplete borders were excluded from analysis.

### Quantification of serum IL-6 and IL-23 levels, and profiling of serum metabolites by targeted UPLC-MS/MS assay and data analysis

IL-6 and IL-23 levels were measured using LEGENDplex^TM^ Mouse Inflammation Panel (13-plex) (BioLegend).

To compare metabolite signatures in young and aged mice at baseline (mock-treated) and at 7 dpi, ultra-high pressure liquid chromatography-tandem mass spectrometry (UPLC-MS/MS) was used. Sera were processed using the MxP^®^ Quant 500 XL kit (BIOCRATES Life Sciences AG, Innsbruck, Austria) as per manufacturer instructions. Briefly, 10 μL of each serum samples (n=5 young mock, n=5 young 7 dpi, n=5 aged mock, and n=5 aged 7 dpi) were loaded onto a filter containing internal standards for normalization. Filters were dried under a stream of nitrogen and a 5% solution of phenyl-isothiocyanate was added for derivatization. Dried analytes were subsequently extracted with 5 mmol/L ammonium acetate in methanol and analyzed by UPLC-MS/MS. The targeted analysis identified and quantified 1019 metabolites (https://biocrates.com/mxp-quant-500-xl/) detected by MS/MS after UPLC separation and flow injection analysis (FIA) (SCIEX 5500 Triple Quad System, SCIEX, Framingham, MA, USA). Data were recorded using the Analyst software (SCIEX) and transferred to the MetIDQ^TM^ software (BIOCRATES), which was used for further data processing i.e., technical validation, quantification, and data export. For each metabolite, peaks were quantified using area-under-the-curves. The analytical method has been fully validated by the kit’s manufacturer according to FDA and EMA guidelines.

Metabolites with ≥ 40% missing values or values below the limit of detection (LOD) in all groups were excluded during initial data cleaning. Consequently, only metabolites with ≥ 60% of concentration values above the LOD in at least one of the three experimental groups were included for statistical analysis. Any remaining missing values were imputed using 1/5 of the minimum positive value of each variable. Given the metabolic interactions between adipose tissue and gut microbiota metabolism, the metabolomic analysis was performed using all metabolites retained after data processing, including lipids. At the end, 552 molecules (that include 11 acylcarnitines, 12 amino acids, 18 amino acid-related metabolites, 6 bile acids, 1 biogenic amine, 4 carboxylic acids, 10 ceramides, 15 cholesterol esters, 1 cresol, 3 diacylglycerols, 4 fatty acids, 12 glycosylceramides, 1 indole derivative, 5 monoacylglycerols, 2 nucleobase-related metabolites, 22 phosphatidic acids, 86 phosphatidylcholines, 93 phosphatidylethanolamines, 51 phosphatidylglycerols, 37 phosphatidylinositols, 15 phosphatidylserines, 4 sphingoid bases, 14 sphingomyelins, 1 sugar and 124 triacylglycerols) were analyzed. Data were log-transformed and analyzed using MetaboAnalyst 5.0 (https://www.metaboanalyst.ca). Lipid analysis was performed using the Lipid Pathway Enrichment Analysis (LIPEA) tool (https://lipea.biotec.tu-dresden.de.).

Statistical comparisons were conducted using the limma package in Phantasus^103^;https://genome.ifma.ru/phantasus Heatmaps, hierarchical clustering, and k-means clustering were performed in Phantasus, with statistical significance set at fold change > 2 and *P* < 0.05.

### Gut microbiota analysis by 16S rRNA gene sequencing

Biomnigene (https://www.biomnigene.fr/fr/, Besançon, France) conducted the DNA extraction from frozen caecal samples using the E.Z.N.A^®^ Stool DNA kit (Omega Bio-tek, Norcross, GA, USA). PCR reactions to amplify the V3-V4 hypervariable region of the 16S rRNA gene were performed using the AccuStartTM II PCR SuperMix (VWR International, Radnor, PA, USA). PCR products were analyzed using a QIAxcel DNA High Resolution Cartridge (QIAGEN). For preparation of libraries, PCR product concentrations were determined using Qubit 4.0, and samples were pooled equimolarly before purification by electrophoresis on PippinHT using a 1.5% agarose cassette (Sage Sciences, Beverly, MA, USA). Sequencing of V3-V4 amplicons was performed on MiSeq Illumina in 2X251 bp by using the Illumina MiSeq Reagent Kit v2 (500 cycles) (Illumina, San Diego, CA, USA). Alpha diversity indices (number of observed OTUs and Shannon diversity index) were computed using QIIME v1.9.1. Gene and metabolic pathway abundances were inferred using PICRUSt2 (Phylogenetic Investigation of Communities by Reconstruction of Unobserved States) software (2020), based on the 16S rRNA gene sequencing results obtained in QIIME 2^104^. Comparison of gut microbiota functional abundances were done based on predicted MetaCyc pathways^105-107^.

### Statistical analyses

Statistical analyses were performed using GraphPad Prism v9 software. A Mann-Whitney test was used to compare two groups (age group comparisons, and mock vs. infected group comparisons), unless otherwise indicated. Differences in relative abundance of individual taxa between groups, were assessed for significance using the Mann-Whitney test controlling for false-discovery rate (FDR) in QIIME v1.9.1. The Wilcoxon signed-rank test (paired *t* test) was used for 16S-rRNA-sequencing analysis of caecal samples. Spearman correlation analysis was performed in R.

## Supplementary Information

Supplementary Information 1.

Supplementary Information 2.

Supplementary Information 3.

Supplementary Information 4.

Supplementary Information 5.

Supplementary Information 6.

Supplementary Information 7.

Supplementary Information 8.

Supplementary Information 9.

Supplementary Information 10.

Supplementary Information 11.

Supplementary Information 12.

Supplementary Information 13.

Supplementary Information 14.

Supplementary Information 15.

Supplementary Information 16.

Supplementary Information 17.

Supplementary Information 18.

Supplementary Information 19.

Supplementary Information 20.

## Data Availability

16S rRNA sequencing data (gut microbiome) are available in the Recherche Data Gouv repository, [10.57745/VNV5J0]. Metabolomic data are available in the Recherche Data Gouv repository, [10.57745/Z71CGC].
