# Supplementary material for: Impact of aging on gut-lung-adipose tissue interactions and lipid metabolism during influenza infection in mice
Source: Sci Rep. 2025 Oct 27;15:37414. doi: 10.1038/s41598-025-21363-1 (PMC12559434; doi:10.1038/s41598-025-21363-1)
Supplement: Supplementary file 1 — Supplementary Information 1. [file 41598_2025_21363_MOESM1_ESM.pdf]

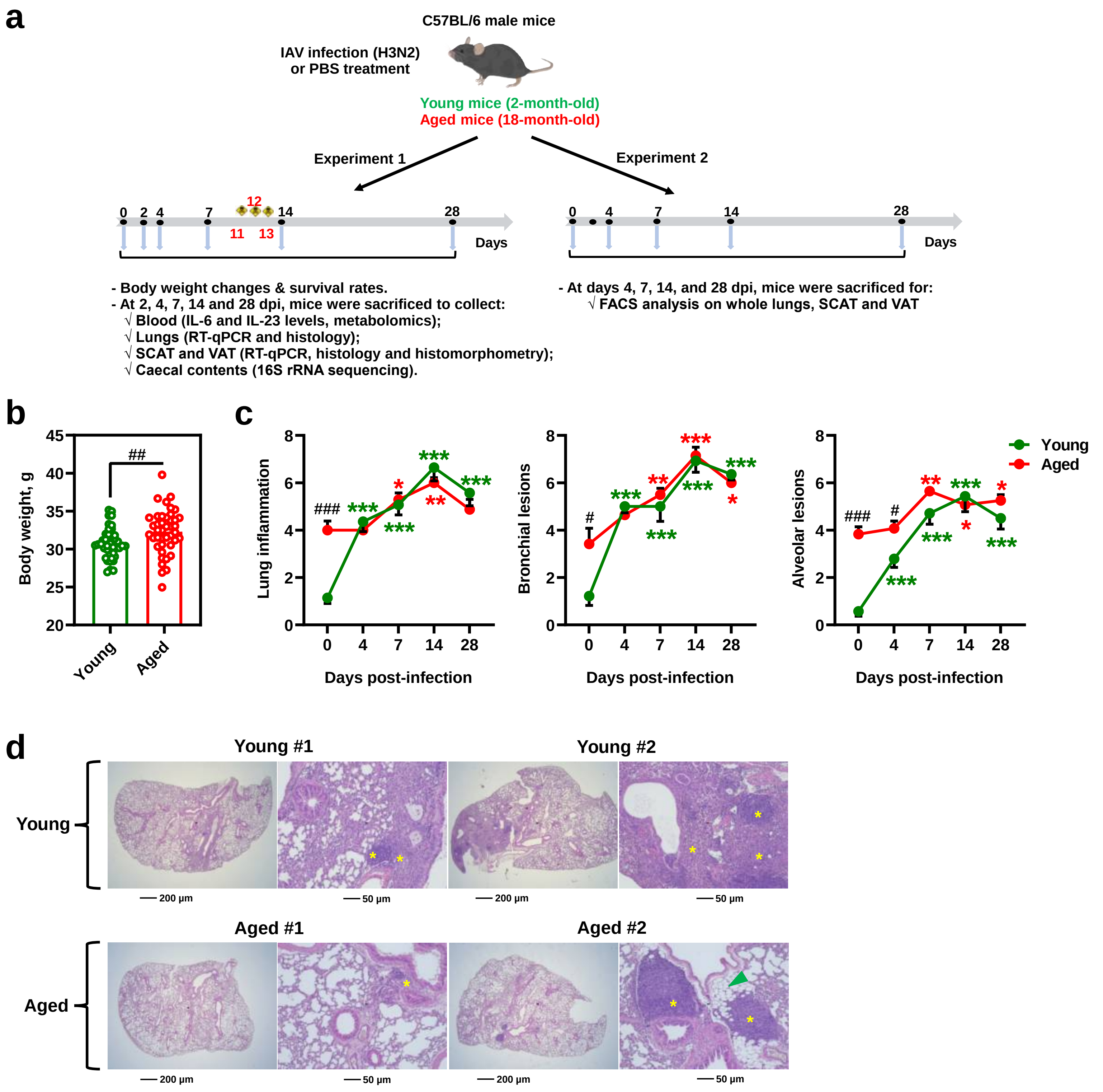
a
C57BL/6 male mice
IAV infection (H3N2)
or PBS treatment
Young mice (2-month-old)
Aged mice (18-month-old)
Experiment 1
Experiment 2
0 2 4 7 11 12 13 14 28
Days
0 4 7 14 28
Days
- Body weight changes & survival rates.
- At 2, 4, 7, 14 and 28 dpi, mice were sacrificed to collect:
√ Blood (IL-6 and IL-23 levels, metabolomics);
√ Lungs (RT-qPCR and histology);
√ SCAT and VAT (RT-qPCR, histology and histomorphometry);
√ Caecal contents (16S rRNA sequencing).
- At days 4, 7, 14, and 28 dpi, mice were sacrificed for:
√ FACS analysis on whole lungs, SCAT and VAT
b
Body weight, g
##
Young
Aged
c
Lung inflammation
Bronchial lesions
Alveolar lesions
Days post-infection
Young
Aged
d
Young #1
Young #2
Aged #1
Aged #2
Young
Aged
200 µm
50 µm

Supplementary Figure 1 – Influenza infection is more severe in lungs of aged mice.

(a) Study design: Young-adult (2-month-old, designated as "young") and aged (18-month-old) male C57BL/6 mice were intranasally administered either 50 PFUs of influenza A virus (H3N2 subtype) or PBS. Two independent infection experiments were conducted.

In the first experiment, 42 mice per age group were used, with 35 mice per group infected. Body weight was recorded daily from 0 to 28 days post-infection (dpi). All infected young mice survived, while three aged mice died at 11, 12, and 13 dpi. At 0, 2, 4, 7, 14 and 28 dpi, seven mice per age group were sacrificed (except at 28 dpi for aged mice, n=4) for collection of lungs, subcutaneous (inguinal) adipose tissue (SCAT), visceral (epidydimal) adipose tissue (VAT), blood, and caecal contents. These samples were analyzed for gene expression, histology, quantitative histomorphometry, serum cytokine quantification, 16S rRNA sequencing, and serum metabolite analyses. Caecal contents were not collected at 2 dpi. In the second experiment, designed for flow cytometry analysis of immune cell populations in lungs, SCAT and VAT, 35 mice per age group were used, with 28 mice per group infected. At 0, 4, 7, 14, and 28 dpi, 7 animals per group were sacrificed (except aged mice at 28 dpi, n=4) for tissue collection and subsequent flow cytometry analysis. Created with BioRender.com. **(b)** Body weight (g) of non-infected young (n=42) and aged (n=42) mice. **(c)** Histopathological subscoring (inflammation, bronchial lesions and alveolar lesions) of lung tissues at 0, 4, 7, 14, and 28 dpi (n=7 per group at each time point, except for aged mice at 28 dpi (n=4)). **(d)** Representative photomicrographs of lung tissues (H&E staining) from two young mice and two aged mice at 28 dpi. Yellow stars: inflammation areas. The presence of adipocyte-like cells was noticed (Green arrows). For **b** and **c**: Data are expressed as mean ± SEM, and were analyzed using a two-sided Mann-Whitney test, with $^{\#}$ indicating *P* values for young vs. aged group comparisons ($^{\#}P < 0.05$, , $^{\#\#}P < 0.01$, $^{\#\#\#}P < 0.001$) and * indicating *P* values for Mock vs. infected group comparisons ($^{*}P < 0.05$, $^{**}P < 0.01$, $^{***}P < 0.0001$). $P <$ 0.05 was considered statistically significant.
