# Supplementary material for: Impact of aging on gut-lung-adipose tissue interactions and lipid metabolism during influenza infection in mice
Source: Sci Rep. 2025 Oct 27;15:37414. doi: 10.1038/s41598-025-21363-1 (PMC12559434; doi:10.1038/s41598-025-21363-1)
Supplement: Supplementary file 2 — Supplementary Information 2. [file 41598_2025_21363_MOESM2_ESM.pdf]

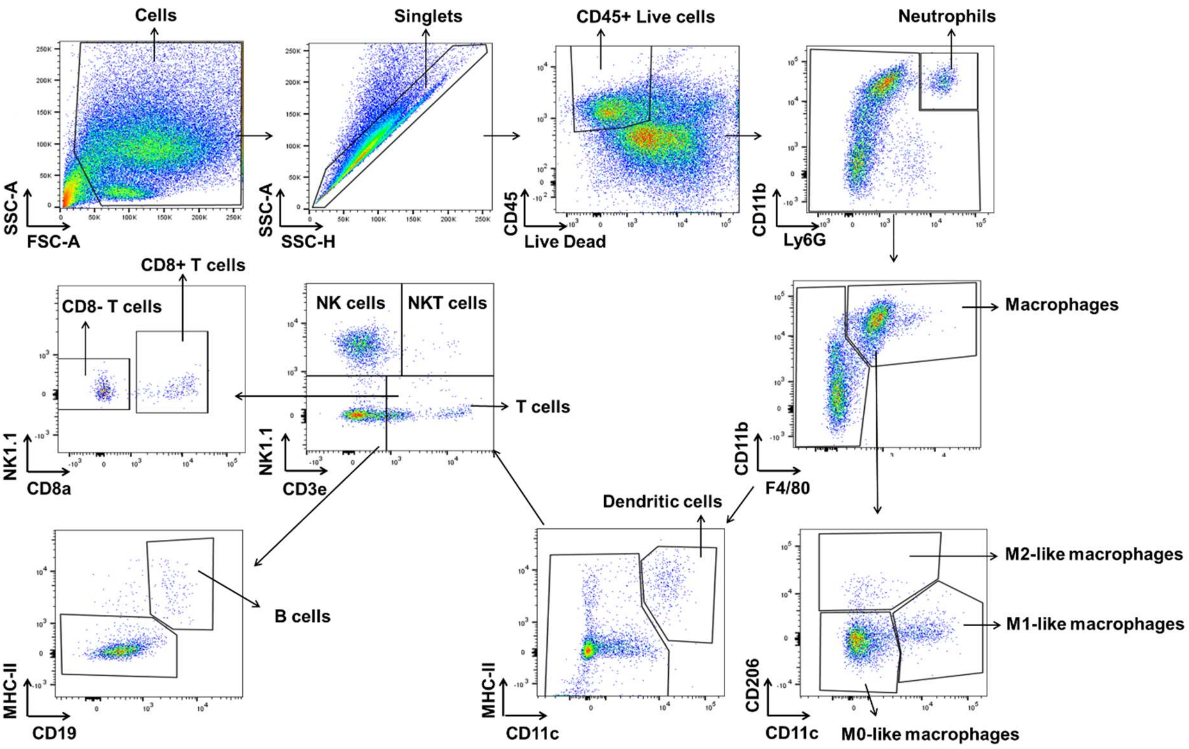
Cells
Singlets
CD45+ Live cells
Neutrophils
SSC-A
FSC-A
SSC-A
SSC-H
CD45
Live Dead
CD11b
Ly6G
CD8+ T cells
CD8- T cells
NK cells
NKT cells
Macrophages
T cells
NK1.1
CD8a
NK1.1
CD3e
CD11b
F4/80
Dendritic cells
M2-like macrophages
B cells
M1-like macrophages
MHC-II
CD19
MHC-II
CD11c
CD206
CD11c
M0-like macrophages

Supplementary Figure 10 – Flow cytometry gating strategy for WAT.

Gating strategy applied to SCAT and VAT (the study design is presented Supplementary Fig. 1a, experiment 2) (gating done on cells from young mice, VAT, IAV-infected (4 dpi)). First, a size gate was applied to select hematopoietic cells, followed by gating for single cells and viability (Zombie Red negativity) and CD45 expression to exclude dead cells and $CD45^-$ cells. Subsequently, cells were gated to identify neutrophils, macrophages (including M1-like, M2-like, and M0-like), dendritic cells, NK cells, NKT cells, $CD3^+$ T cells, $CD8^+$ T cells, $CD8^-$ T cells, and B cells.
