# Supplementary material for: Impact of aging on gut-lung-adipose tissue interactions and lipid metabolism during influenza infection in mice
Source: Sci Rep. 2025 Oct 27;15:37414. doi: 10.1038/s41598-025-21363-1 (PMC12559434; doi:10.1038/s41598-025-21363-1)
Supplement: Supplementary file 3 — Supplementary Information 3. [file 41598_2025_21363_MOESM3_ESM.pdf]

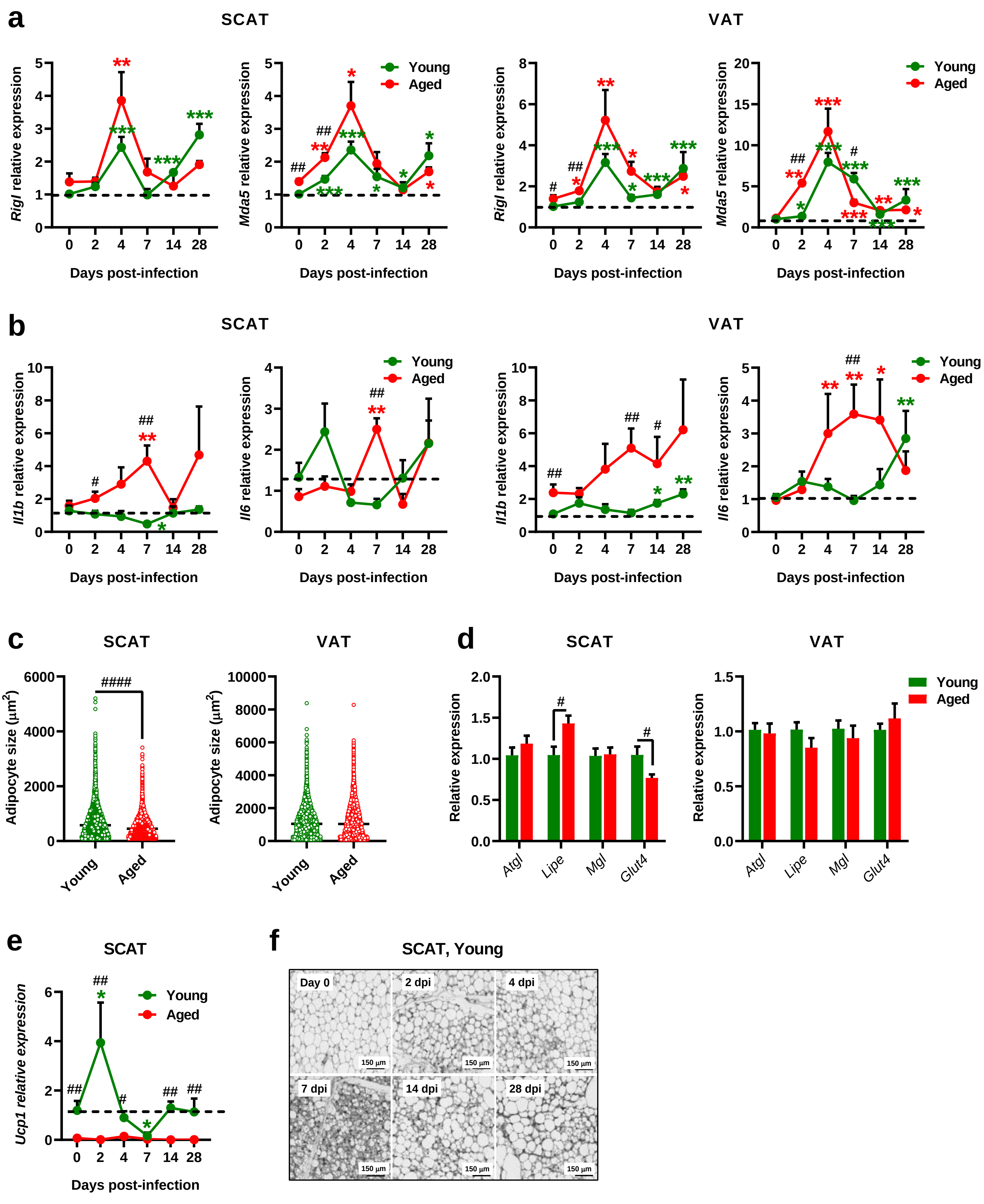
a
SCAT
VAT
Rigl relative expression
Mda5 relative expression
Young
Aged
Days post-infection
0 2 4 7 14 28
b
SCAT
VAT
Il1b relative expression
Il6 relative expression
Young
Aged
Days post-infection
c
SCAT
VAT
Adipocyte size (μm²)
####
Young
Aged
d
SCAT
VAT
Relative expression
Young
Aged
Atgl
Lipe
Mgl
Glut4
e
SCAT
Ucp1 relative expression
Young
Aged
Days post-infection
f
SCAT, Young
Day 0
2 dpi
4 dpi
7 dpi
14 dpi
28 dpi
150 μm

Supplementary Figure 2 – Influenza infection differentially impacts the WAT of young mice and aged mice.

(a) mRNA expression levels of *RigI* and *Mda5* in SCAT (left) and VAT (right) at 0, 2, 4, 7, 14, and 28 dpi, n=7 per group at each time point except for aged mice at 28 dpi (n=4). **(b)** mRNA expression levels of *Il1b* and *Il6* in SCAT (left) and VAT (right) at 0, 2, 4, 7, 14, and 28 dpi, n=7 per group at each time point except for aged mice at 28 dpi (n=4). **(c)** Superplots showing the size of individual adipocytes, as well as the mean values (μm^2) in the SCAT (left) and VAT (right) from mock-treated young (n=4) mice and aged (n=4) mice. **(d)** mRNA expression levels of the lipolysis-related genes *Atgl* (encoding adipose triglyceride lipase), *Lipe* encoding hormone sensitive lipase), and *Mgl* (encoding monoglyceride lipase), and the lipogenic gene *Glut4* (encoding glucose transporter 4) in the SCAT (left) and VAT (right) from mock-treated young (n=7) mice and aged (n=7) mice. **(e)** mRNA expression levels of *Ucp1* (encoding mitochondrial uncoupling protein 1) in SCAT at 0, 2, 4, 7, 14, and 28 dpi, n=7 per group at each time point except for aged mice at 28 dpi (n=4). **(f)** Representative H&E-stained photomicrographs of the SCAT from young mice at days 0, 2, 4, 7, 14, and 28 dpi. Clusters of small, multilocular brown-like adipocytes appear in young SCAT as early as 2 dpi, reaching a peak at 7 dpi. For **a**, **b**, **d** and **e**: Data are expressed as mean ± SEM. Relative expression is presented as $2^{-\Delta\Delta CT}$. Data were normalized to *Eef2* housekeeping gene expression levels, and expressed relative to the expression obtained in the samples from mock-treated young mice. Statistical analysis was performed using a two-sided Mann-Whitney test, with # indicating *P* values for age group comparisons ($^{\#}P < 0.05$, $^{\#\#}P < 0.01$, $^{\#\#\#\#}P < 0.0001$) and * indicating *P* values for mock-treated vs. infected group groups ($^{*}P < 0.05$, $^{**}P < 0.01$, $^{***}P < 0.001$). $P < 0.05$ was considered statistically significant.
