# Supplementary material for: Impact of aging on gut-lung-adipose tissue interactions and lipid metabolism during influenza infection in mice
Source: Sci Rep. 2025 Oct 27;15:37414. doi: 10.1038/s41598-025-21363-1 (PMC12559434; doi:10.1038/s41598-025-21363-1)
Supplement: Supplementary file 4 — Supplementary Information 4. [file 41598_2025_21363_MOESM4_ESM.pdf]

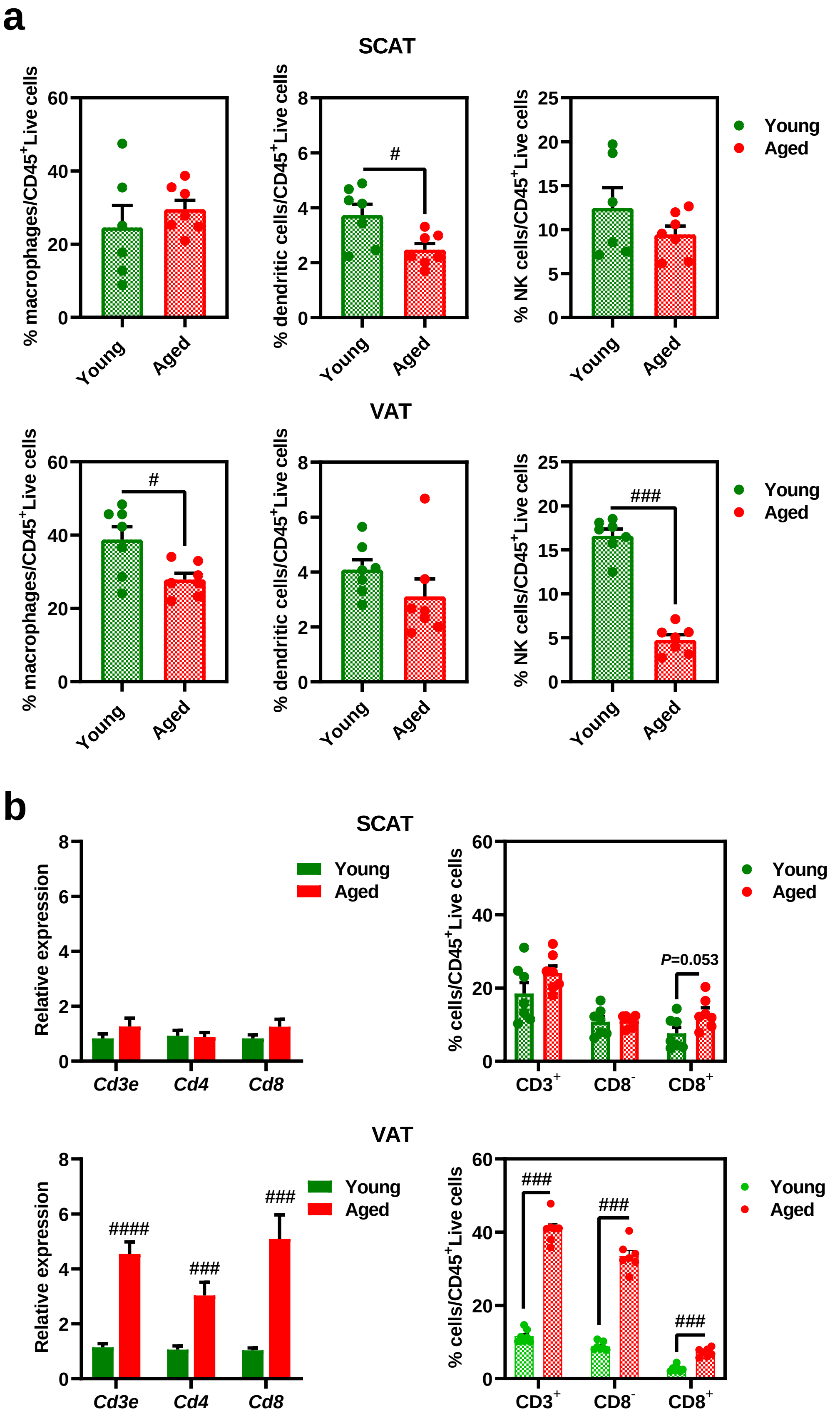

a
SCAT
% macrophages/CD45+Live cells
% dendritic cells/CD45+Live cells
% NK cells/CD45+Live cells
Young
Aged
#
VAT
#
###
b
SCAT
Relative expression
Cd3e
Cd4
Cd8
% cells/CD45+Live cells
CD3+
CD8-
CD8+
P=0.053
VAT
####
###
###

Supplementary Figure 3 – Age-related changes in WAT immune cell composition.

(a) Innate immune cell (macrophages, dendritic cells, NK cells) frequencies among $CD45^+$ Live cells in the SCAT (above) and VAT (below) from mock-treated young (n=7) mice and aged (n=7) mice. **(b)** mRNA expression levels of *Cd3e*, *Cd4*, and *Cd8* in the SCAT (above) and VAT (below) from mock-treated young mice and aged mice (left), and frequencies of $CD3^+$ T cells, $CD8^-$ T cells and $CD8^+$ T cells among $CD45^+$ Live cells in the SCAT (above) and VAT (below) from mock-treated young mice and aged mice (right). Data are expressed as mean ± SEM, n=7 animal per group. For **a**: Macrophages were identified as Live-$Dead^-CD45^+CD11b^+F4/80^+$ cells, dendritic cells as Live-$Dead^-CD45^+Ly6G^-F4/80^-$ $MHCII^{hi}CD11c^{hi}$ cells, and NK cells as Live-$Dead^-CD45^+Ly6G^-F4/80^-NK1.1^+$ $CD3e^-$ cells (refer to gating strategy Supplementary Fig. 10), individual values are shown. For **b**: Relative expression is presented as $2^{-\Delta\Delta CT}$. Data were normalized to *Eef2* housekeeping gene expression, and expressed relative to the expression obtained in the samples from mock-treated young mice. Statistical analysis was performed using a two-sided Mann-Whitney test, with $^{\#}$ indicating *P* values for age group comparisons ($^{\#}P < 0.05$, $^{\#\#\#}P < 0.001$, $^{\#\#\#\#}P < 0.0001$). $P < 0.05$ was considered statistically significant.
