# Supplementary material for: Impact of aging on gut-lung-adipose tissue interactions and lipid metabolism during influenza infection in mice
Source: Sci Rep. 2025 Oct 27;15:37414. doi: 10.1038/s41598-025-21363-1 (PMC12559434; doi:10.1038/s41598-025-21363-1)
Supplement: Supplementary file 5 — Supplementary Information 5. [file 41598_2025_21363_MOESM5_ESM.pdf]

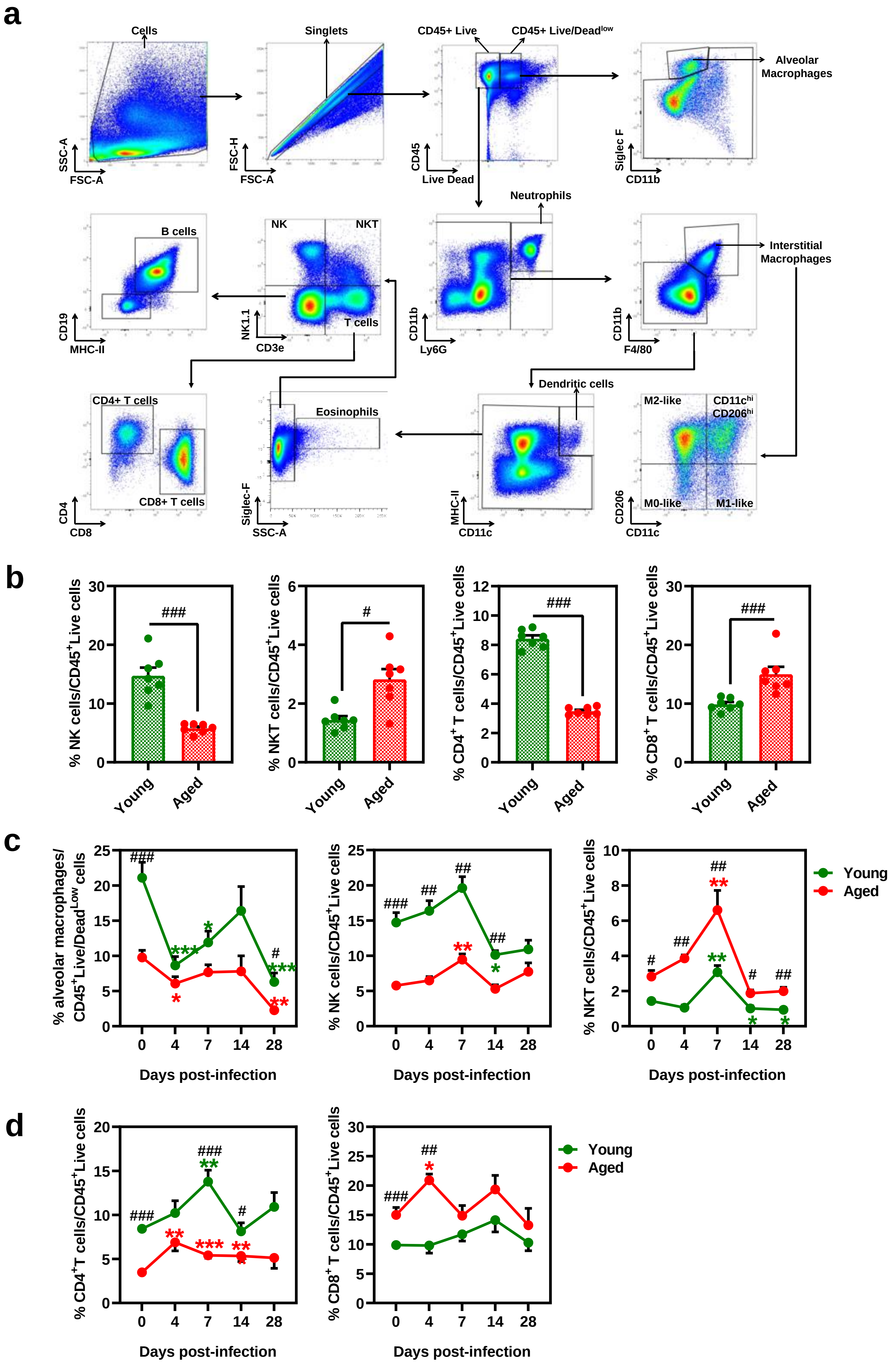
a
Cells
SSC-A
FSC-A
Singlets
FSC-H
FSC-A
CD45+ Live
CD45+ Live/Dead^low
CD45
Live Dead
Alveolar Macrophages
Siglec F
CD11b
Neutrophils
CD11b
Ly6G
Interstitial Macrophages
CD11b
F4/80
NK
NKT
T cells
NK1.1
CD3e
B cells
CD19
MHC-II
CD4+ T cells
CD8+ T cells
CD4
CD8
Eosinophils
Siglec-F
SSC-A
Dendritic cells
MHC-II
CD11c
M2-like
CD11c^hi CD206^hi
M0-like
M1-like
CD206
CD11c
b
% NK cells/CD45+Live cells
% NKT cells/CD45+Live cells
% CD4+ T cells/CD45+Live cells
% CD8+ T cells/CD45+Live cells
###
#
###
###
Young
Aged
c
% alveolar macrophages/ CD45+Live/Dead^Low cells
% NK cells/CD45+Live cells
% NKT cells/CD45+Live cells
Days post-infection
Young
Aged
d
% CD4+T cells/CD45+Live cells
% CD8+ T cells/CD45+Live cells
Days post-infection
Young
Aged

Supplementary Figure 4 – Impact of aging and influenza infection on lung immune cell composition.

(a) Gating strategy applied to lung tissue (the study design is presented Supplementary Fig. 1a, experiment 2) (gating done on cells from young mice, mock-treated). **(b)** NK cell, NKT cell, $CD4^+$ T cell, and $CD8^+$ T cell frequencies among $CD45^+$ Live cells in lungs from mock-treated young (n=7) and aged (n=7) mice. **(c)** Alveolar macrophage frequencies among $CD45^+$ Live-Dead^{low} cells, and NK cell and NKT cell frequencies among $CD45^+$ Live cells in lungs from young and aged mice at 0, 4, 7, 14, and 28 dpi. **(d)** $CD4^+$ T cell and $CD8^+$ T cell frequencies among $CD45^+$ Live cells in lungs from young and aged mice at 0, 4, 7, 14, and 28 dpi. Data are expressed as mean ± SEM. For **c** and **d**: n=7 mice per group at each time point, except for aged mice at 28 dpi (n=4). Groups were compared using a two-sided Mann-Whitney test, with $^{\#}$ indicating P values for young vs. aged group comparisons ($^{\#}P < 0.05$, $^{\#\#}P < 0.01$, $^{\#\#\#}P < 0.001$), and * indicating P values for mock vs. infected group comparisons ($^{*}P < 0.05$, $^{**}P < 0.01$, $^{***}P < 0.001$). $P < 0.05$ was considered statistically significant.
