# Supplementary material for: Impact of aging on gut-lung-adipose tissue interactions and lipid metabolism during influenza infection in mice
Source: Sci Rep. 2025 Oct 27;15:37414. doi: 10.1038/s41598-025-21363-1 (PMC12559434; doi:10.1038/s41598-025-21363-1)
Supplement: Supplementary file 6 — Supplementary Information 6. [file 41598_2025_21363_MOESM6_ESM.pdf]

	Young					Aged				
	**Mock**	**4 dpi**	**7 dpi**	**14 dpi**	**28 dpi**	**Mock**	**4 dpi**	**7 dpi**	**14 dpi**	**28 dpi**
Neutrophils	14	24.7	11.1	14.5^{*}	12.8	13.2	7.8	9.3	5.6^{**}	$3.7^{**, \#\#}$
Eosinophils	1.1	0.3^{***}	0.5^{**}	0.2^{**}	0.2^{***}	0.9	0.6	0.8	0.1^{***}	0.2^{**}
Interstitial Mφ	8.4	9.1	11.8	17.4^{**}	7.5	9.8	$4.9^{**, \#\#}$	11	13.3^{*}	6.4
Alveolar Mφ	21.1	8.7^{***}	11.9^{*}	16.4	6.3^{***}	$9.8^{\#\#\#\#}$	6.1^{*}	7.7	7.8	$2.3^{**,\#}$
M1-like Mφ	0.3	0.9	1.2	3.8^{***}	1.1	0.3	0.5	1.1	3.6^{***}	0.3
M2-like Mφ	5.5	4.1	5.1	2.2^{***}	2.3^{*}	6.6	$2.3^{**, \#}$	5.4	$1^{***, \#}$	2.4^{**}
M0-like Mφ	0.6	1.9	3.4^{*}	9.9^{***}	3.6^{**}	0.9	1.1	2.6	7.6^{***}	2.5^{*}
Dendritic cells	0.5	0.3^{**}	0.6	0.6	0.4^{*}	0.5	0.3^{**}	0.7^{**}	0.4	0.4
NK cells	14.7	16.4	19.6	10.1^{*}	10.9	$5.8^{\#\#\#\#}$	$6.5^{\#\#}$	$9.4^{**, \#\#}$	$5.3^{\#\#}$	7.7
NKT cells	1.4	1.1	3.1^{**}	1^{*}	0.9^{*}	$2.8^{\#}$	$3.9^{\#\#}$	$6.6^{**, \#\#}$	$1.9^{\#}$	$2^{\#\#}$
B cells	33.1	19^{*}	16^{*}	22.4	30.7	40.1	$40.1^{\#\#}$	28.3	$33.1^{\#}$	43.9
T cells	19.1	21.7	27.2^{**}	23.2	26	20.1	28.8*	22.5	25.4	23.4
$CD4^{+}$ T cells	8.4	10.2	13.8^{**}	8.1	10.9	$3.5^{\#\#\#}$	6.9^{**}	$5.4^{***, \#\#\#}$	$5.3^{**, \#}$	5.1
$CD8^{+}$ T cells	9.9	9.8	11.7	14.1	10.3	$15^{\#\#\#}$	$20.9^{*, \#\#}$	14.9	19.4	13.2

Supplementary Table 3 – Frequency of immune cells among CD45$^+$ Live cells in lungs from young and aged mice during influenza infection.

Immune cells were characterized in lungs by flow cytometry (refer to Gating strategy Supplementary Fig. 4a). Data are expressed as mean ± SEM, n=7 mice per group at each time point, except foraged mice at 28 dpi (n=4). Groups were compared using a two-sided Mann-Whitney test, with $^{\#}$ indicating *P* values for age group ($^{\#}P < 0.05$, $^{\#\#}P < 0.01$, $^{\#\#\#}P < 0.001$), and * indicating *P* values for mock vs. infected group comparisons ($^{*}P < 0.05$, $^{**}P < 0.01$, $^{***}P < 0.001$). $P < 0.05$ was considered statistically significant.

Mφ : macrophages.
