# Supplementary material for: Impact of aging on gut-lung-adipose tissue interactions and lipid metabolism during influenza infection in mice
Source: Sci Rep. 2025 Oct 27;15:37414. doi: 10.1038/s41598-025-21363-1 (PMC12559434; doi:10.1038/s41598-025-21363-1)
Supplement: Supplementary file 9 — Supplementary Information 9. [file 41598_2025_21363_MOESM9_ESM.pdf]

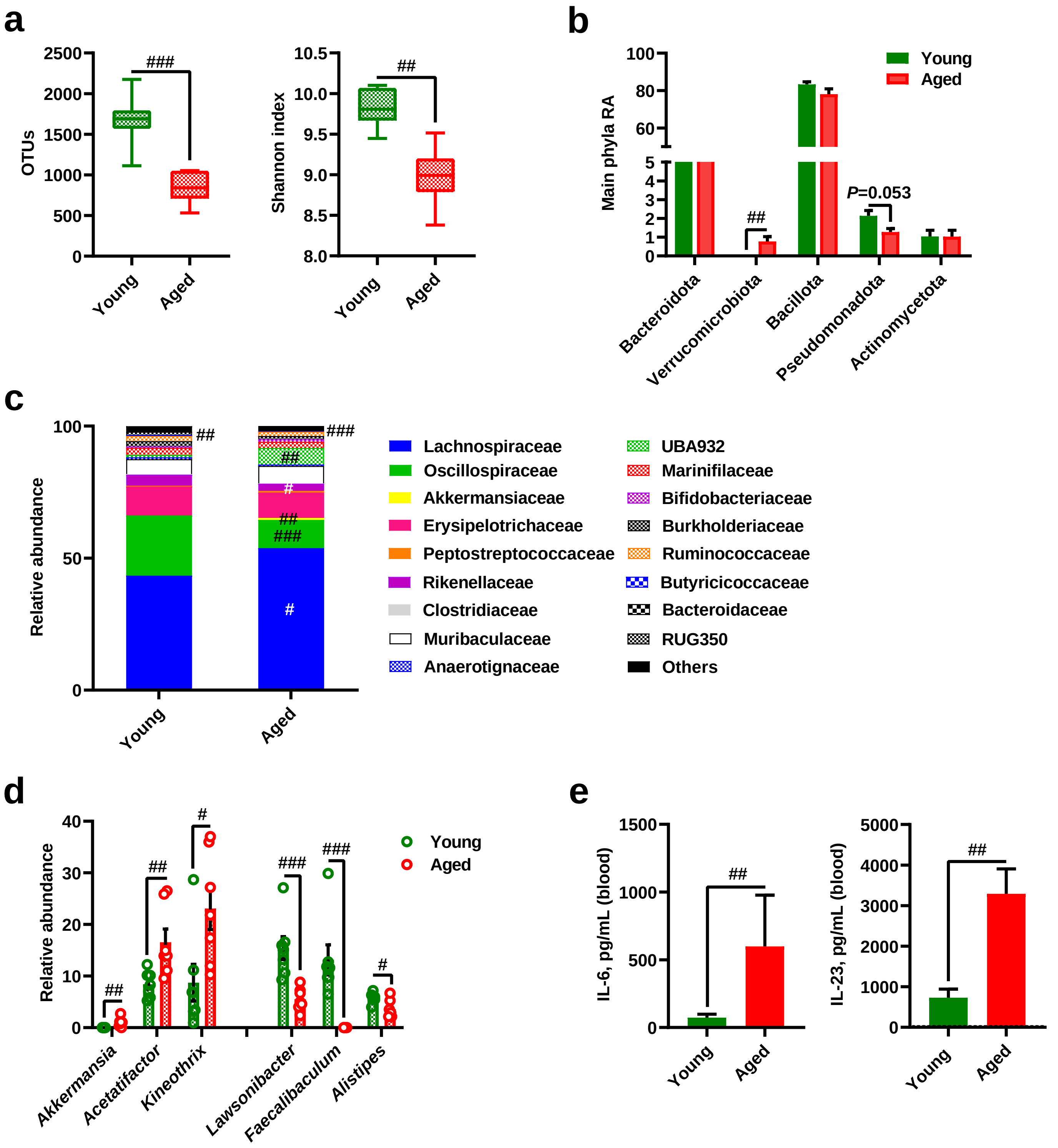
a
2500
2000
1500
1000
500
0
OTUs
###
Young
Aged
10.5
10.0
9.5
9.0
8.5
8.0
Shannon index
##
Young
Aged
b
100
80
60
5
4
3
2
1
0
Main phyla RA
Young
Aged
##
P=0.053
Bacteroidota
Verrucomicrobiota
Bacillota
Pseudomonadota
Actinomycetota
c
100
50
0
Relative abundance
##
###
##
#
##
###
#
Young
Aged
Lachnospiraceae
Oscillospiraceae
Akkermansiaceae
Erysipelotrichaceae
Peptostreptococcaceae
Rikenellaceae
Clostridiaceae
Muribaculaceae
Anaerotignaceae
UBA932
Marinifilaceae
Bifidobacteriaceae
Burkholderiaceae
Ruminococcaceae
Butyricicoccaceae
Bacteroidaceae
RUG350
Others
d
40
30
20
10
0
Relative abundance
##
##
#
###
###
#
Young
Aged
Akkermansia
Acetatifactor
Kineothrix
Lawsonibacter
Faecalibaculum
Alistipes
e
1500
1000
500
0
IL-6, pg/mL (blood)
##
Young
Aged
5000
4000
3000
2000
1000
0
IL-23, pg/mL (blood)
##
Young
Aged

Supplementary Figure 5 – Aging induced changes in gut microbiota diversity and composition. **(a)** Alpha diversity metrics. Boxplots illustrate the comparison of species richness (OTUs) and species diversity (Shannon diversity index) between the gut microbiota of mock-treated young and aged mice. **(b)** Relative abundances (RA) of the main phyla (*Bacteroidota*, *Verrucomicrobiota*, *Bacillota*, *Pseudomonadota* and *Actinomycetota*) in the gut microbiota of mock-treated young and aged mice. **(c)** Relative abundances of bacterial communities at the family level in the gut microbiota of mock-treated young and aged mice. Main families are represented, with remaining families grouped under "others". **(d)** Relative abundances of *Akkermansia*, *Acetatifactor*, *Kineothrix*, *Lawsonibacter*, *Faecalibaculum* and *Alistipes* genera in the gut microbiota of mock-treated young and aged mice. **(e)** IL-6 and IL-23 concentrations (pg/mL) in the blood of mock-treated young and aged mice. Data are expressed as mean ± SEM, n=7 animals per group. Statistical analysis was performed using a two-sided Mann-Whitney test, with $^{\#}$ indicating P values for age group comparisons ($^{\#}P < 0.05$, $^{\#\#}P < 0.01$, $^{\#\#\#}P < 0.001$). $P < 0.05$ was considered statistically significant.
