# Supplementary material for: Impact of aging on gut-lung-adipose tissue interactions and lipid metabolism during influenza infection in mice
Source: Sci Rep. 2025 Oct 27;15:37414. doi: 10.1038/s41598-025-21363-1 (PMC12559434; doi:10.1038/s41598-025-21363-1)
Supplement: Supplementary file 10 — Supplementary Information 10. [file 41598_2025_21363_MOESM10_ESM.pdf]

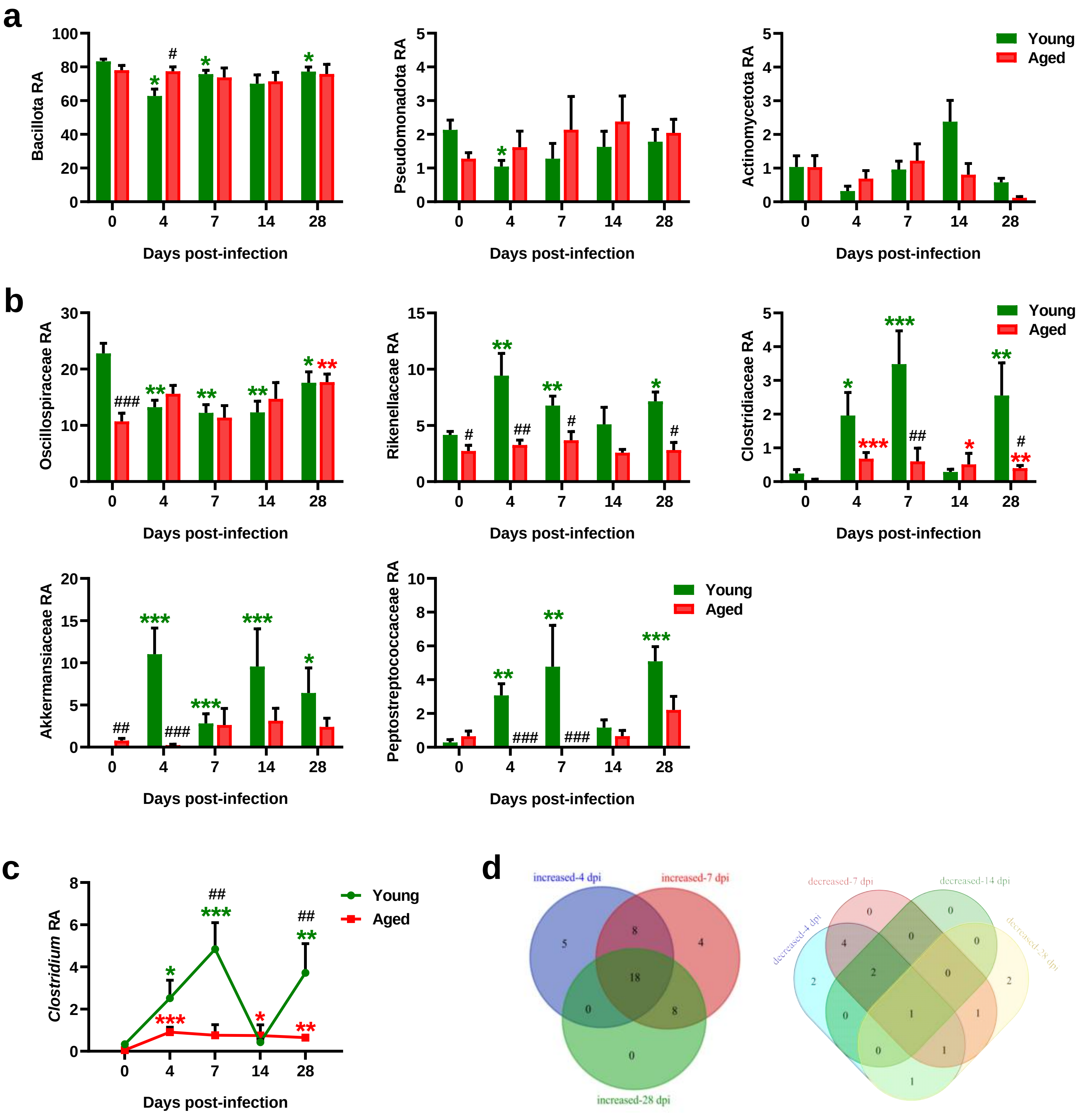
a
Bacillota RA
Pseudomonadota RA
Actinomycetota RA
Young
Aged
Days post-infection
b
Oscillospiraceae RA
Rikenellaceae RA
Clostridiaceae RA
Akkermansiaceae RA
Peptostreptococcaceae RA
Young
Aged
Days post-infection
c
Clostridium RA
Young
Aged
Days post-infection
d
increased-4 dpi
increased-7 dpi
increased-28 dpi
decreased-4 dpi
decreased-7 dpi
decreased-14 dpi
decreased-28 dpi

Supplementary Figure 6 – Influenza infection induced more changes in the gut microbiota from younger adult mice.

(a) Relative abundances (RA) of *Bacillota*, *Pseudomonadota* and *Actinomycetota* phyla in the gut microbiota of mock-treated and infected young and aged mice. **(b)** RA of *Oscillospiraceae*, *Rikenellaceae*, *Clostridiaceae*, *Akkermansiaceae* and *Peptostreptococcaceae* families in the gut microbiota of mock-treated and infected young and aged mice. **(c)** *Clostridium* genus RA in mock-treated and infected young and aged mice. **(d)** Venn diagrams showing increased (left) or decreased (right) KEGG pathways in the gut microbiota of infected young-mice (PICRUSt2 analysis). For **a**, **b** and **c**: Data are expressed as mean ± SEM, n=7 animals per group, except for n=4 aged mice at 28 dpi. Statistical analysis was performed using a two-sided Mann-Whitney test, with $^{\#}$ indicating P values for age group comparisons ($^{\#}P < 0.05$, $^{\#\#}P < 0.01$, $^{\#\#\#}P < 0.001$) and * indicating P values for mock-treated vs. infected group comparisons ($^{*}P < 0.05$, $^{**}P < 0.01$, $^{***}P < 0.0001$). $P < 0.05$ was considered statistically significant.
