# Supplementary material for: Impact of aging on gut-lung-adipose tissue interactions and lipid metabolism during influenza infection in mice
Source: Sci Rep. 2025 Oct 27;15:37414. doi: 10.1038/s41598-025-21363-1 (PMC12559434; doi:10.1038/s41598-025-21363-1)
Supplement: Supplementary file 11 — Supplementary Information 11. [file 41598_2025_21363_MOESM11_ESM.pdf]

a

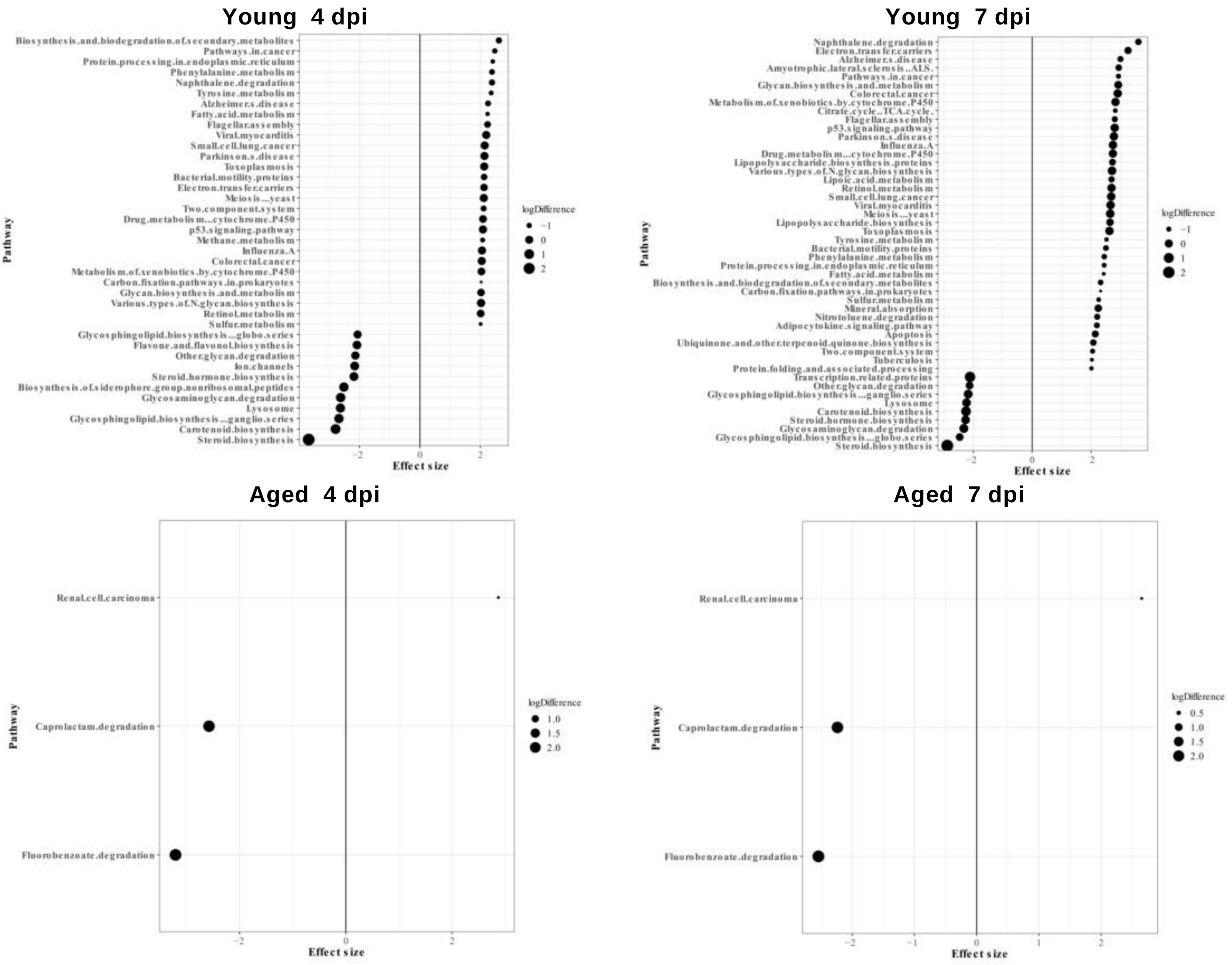
Young 4 dpi
Young 7 dpi
Aged 4 dpi
Aged 7 dpi
Pathway
Effect size
logDifference
Renal.cell.carcinoma
Caprolactam.degradation
Fluorobenzoate.degradation
Steroid.biosynthesis

b

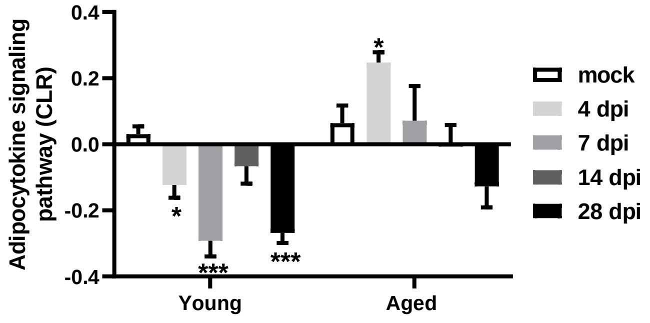
Adipocytokine signaling pathway (CLR)
0.4
0.2
0.0
-0.2
-0.4
Young
Aged
mock
4 dpi
7 dpi
14 dpi
28 dpi
*
***
***
*

Supplementary Figure 7 – Influenza infection's impact of gut bacterial functionalities.

(a) Functional categories encoded by the gut microbiota: differential enrichment of KEGG functions in infected young mice (above) and aged mice (below) at 4 dpi (left) and 7 dpi (right) (PICRUSt2 analysis). (**b**) KEGG Adipocytokine signaling pathway in young and aged mice at 0, 4, 7, 14 and 28 dpi. For **b**: Data are expressed as mean ± SEM, n=7 animals per group, except for n=4 aged mice at 28 dpi. Statistical analysis was performed using a two-sided Mann-Whitney test, with * indicating P values for mock-treated vs. infected group comparisons ($^{*}P < 0.05$, $^{***}P < 0.0001$). $P < 0.05$ was considered statistically significant.
