# Supplementary material for: Impact of aging on gut-lung-adipose tissue interactions and lipid metabolism during influenza infection in mice
Source: Sci Rep. 2025 Oct 27;15:37414. doi: 10.1038/s41598-025-21363-1 (PMC12559434; doi:10.1038/s41598-025-21363-1)
Supplement: Supplementary file 12 — Supplementary Information 12. [file 41598_2025_21363_MOESM12_ESM.pdf]

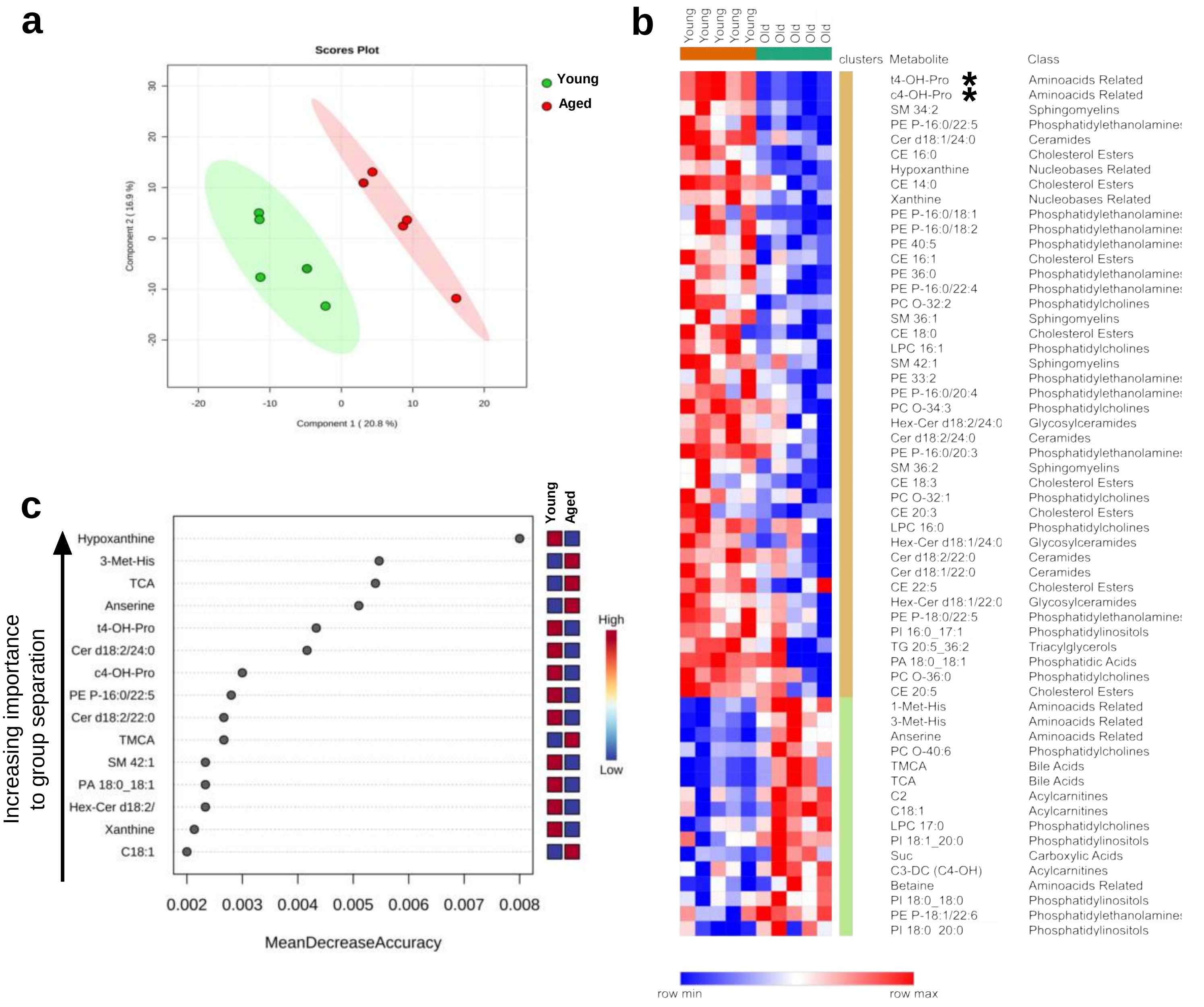
a
Scores Plot
Young
Aged
Component 2 (16.9 %)
Component 1 (20.8 %)
-20
-10
0
10
20
30
b
Young
Young
Young
Young
Young
Old
Old
Old
Old
Old
clusters
Metabolite
Class
t4-OH-Pro *
c4-OH-Pro *
SM 34:2
PE P-16:0/22:5
Cer d18:1/24:0
CE 16:0
Hypoxanthine
CE 14:0
Xanthine
PE P-16:0/18:1
PE P-16:0/18:2
PE 40:5
CE 16:1
PE 36:0
PE P-16:0/22:4
PC O-32:2
SM 36:1
CE 18:0
LPC 16:1
SM 42:1
PE 33:2
PE P-16:0/20:4
PC O-34:3
Hex-Cer d18:2/24:0
Cer d18:2/24:0
PE P-16:0/20:3
SM 36:2
CE 18:3
PC O-32:1
CE 20:3
LPC 16:0
Hex-Cer d18:1/24:0
Cer d18:2/22:0
Cer d18:1/22:0
CE 22:5
Hex-Cer d18:1/22:0
PE P-18:0/22:5
PI 16:0_17:1
TG 20:5_36:2
PA 18:0_18:1
PC O-36:0
CE 20:5
1-Met-His
3-Met-His
Anserine
PC O-40:6
TMCA
TCA
C2
C18:1
LPC 17:0
PI 18:1_20:0
Suc
C3-DC (C4-OH)
Betaine
PI 18:0_18:0
PE P-18:1/22:6
PI 18:0_20:0
Aminoacids Related
Sphingomyelins
Phosphatidylethanolamines
Ceramides
Cholesterol Esters
Nucleobases Related
Phosphatidylcholines
Glycosylceramides
Phosphatidylinositols
Triacylglycerols
Phosphatidic Acids
Bile Acids
Acylcarnitines
Carboxylic Acids
row min
row max
c
Increasing importance to group separation
Hypoxanthine
3-Met-His
TCA
Anserine
t4-OH-Pro
Cer d18:2/24:0
c4-OH-Pro
PE P-16:0/22:5
Cer d18:2/22:0
TMCA
SM 42:1
PA 18:0_18:1
Hex-Cer d18:2/
Xanthine
C18:1
Young
Aged
High
Low
0.002
0.003
0.004
0.005
0.006
0.007
0.008
MeanDecreaseAccuracy

Supplementary Figure 8 – Age-related changes in serum metabolites.

Serum samples from mock-treated young (n=5) and aged (old) (n=5) mice were analyzed. **(a)** PLS-DA score for metabolite profiling data (PERMANOVA, F-value = 1.6215, R-squared = 0. 16853, *P*-value (based on 999 permutations) = 0.224) . **(b)** Heatmap displaying 58 differential metabolites (*P* < 0.05, linear model) organized into 2 clusters using unsupervised machine learning algorithm K-means. * indicates metabolites with adjusted *P* < 0. 001. Red/blue colors indicate increased/decreased levels. **(c)** Random forest ranking of top-15 predictive metabolites for aging status.
