# Supplementary material for: Impact of aging on gut-lung-adipose tissue interactions and lipid metabolism during influenza infection in mice
Source: Sci Rep. 2025 Oct 27;15:37414. doi: 10.1038/s41598-025-21363-1 (PMC12559434; doi:10.1038/s41598-025-21363-1)
Supplement: Supplementary file 13 — Supplementary Information 13. [file 41598_2025_21363_MOESM13_ESM.pdf]

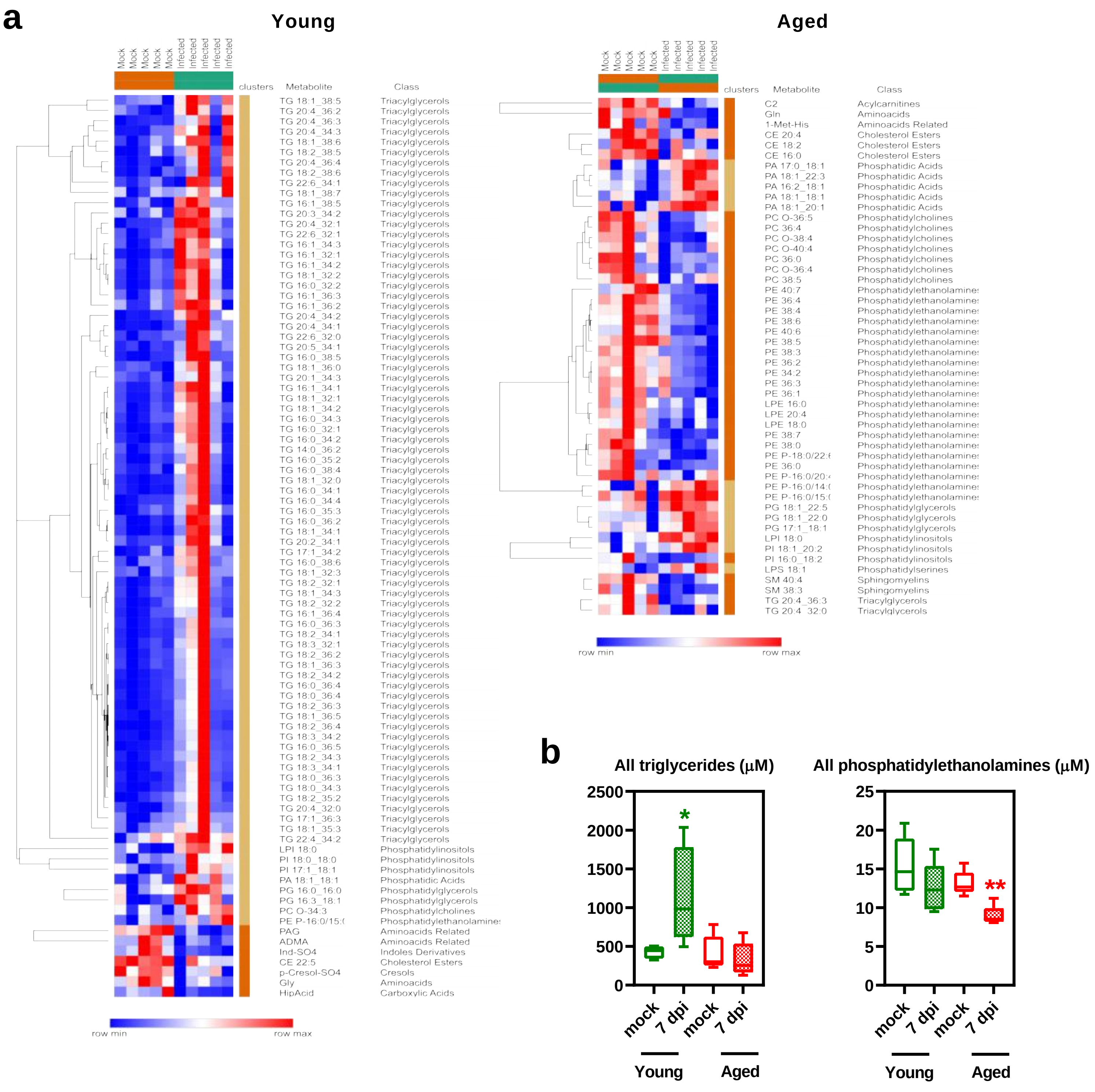
a
Young
Mock
Mock
Mock
Mock
Mock
Infected
Infected
Infected
Infected
Infected
clusters
Metabolite
Class
TG 18:1_38:5 Triacylglycerols
TG 20:4_36:2 Triacylglycerols
TG 20:4_36:3 Triacylglycerols
TG 20:4_34:3 Triacylglycerols
TG 18:1_38:6 Triacylglycerols
TG 18:2_38:5 Triacylglycerols
TG 20:4_36:4 Triacylglycerols
TG 18:2_38:6 Triacylglycerols
TG 22:6_34:1 Triacylglycerols
TG 18:1_38:7 Triacylglycerols
TG 16:1_38:5 Triacylglycerols
TG 20:3_34:2 Triacylglycerols
TG 20:4_32:1 Triacylglycerols
TG 22:6_32:1 Triacylglycerols
TG 16:1_34:3 Triacylglycerols
TG 16:1_32:1 Triacylglycerols
TG 16:1_34:2 Triacylglycerols
TG 18:1_32:2 Triacylglycerols
TG 16:0_32:2 Triacylglycerols
TG 16:1_36:3 Triacylglycerols
TG 16:1_36:2 Triacylglycerols
TG 20:4_34:2 Triacylglycerols
TG 20:4_34:1 Triacylglycerols
TG 22:6_32:0 Triacylglycerols
TG 20:5_34:1 Triacylglycerols
TG 16:0_38:5 Triacylglycerols
TG 18:1_36:0 Triacylglycerols
TG 20:1_34:3 Triacylglycerols
TG 16:1_34:1 Triacylglycerols
TG 18:1_32:1 Triacylglycerols
TG 18:1_34:2 Triacylglycerols
TG 16:0_34:3 Triacylglycerols
TG 16:0_32:1 Triacylglycerols
TG 16:0_34:2 Triacylglycerols
TG 14:0_36:2 Triacylglycerols
TG 16:0_35:2 Triacylglycerols
TG 16:0_38:4 Triacylglycerols
TG 18:1_32:0 Triacylglycerols
TG 16:0_34:1 Triacylglycerols
TG 16:0_34:4 Triacylglycerols
TG 16:0_35:3 Triacylglycerols
TG 16:0_36:2 Triacylglycerols
TG 18:1_34:1 Triacylglycerols
TG 20:2_34:1 Triacylglycerols
TG 17:1_34:2 Triacylglycerols
TG 16:0_38:6 Triacylglycerols
TG 18:1_32:3 Triacylglycerols
TG 18:2_32:1 Triacylglycerols
TG 18:1_34:3 Triacylglycerols
TG 18:2_32:2 Triacylglycerols
TG 16:1_36:4 Triacylglycerols
TG 16:0_36:3 Triacylglycerols
TG 18:2_34:1 Triacylglycerols
TG 18:3_32:1 Triacylglycerols
TG 18:2_36:2 Triacylglycerols
TG 18:1_36:3 Triacylglycerols
TG 18:2_34:2 Triacylglycerols
TG 16:0_36:4 Triacylglycerols
TG 18:0_36:4 Triacylglycerols
TG 18:2_36:3 Triacylglycerols
TG 18:1_36:5 Triacylglycerols
TG 18:2_36:4 Triacylglycerols
TG 18:3_34:2 Triacylglycerols
TG 16:0_36:5 Triacylglycerols
TG 18:2_34:3 Triacylglycerols
TG 18:3_34:1 Triacylglycerols
TG 18:0_36:3 Triacylglycerols
TG 18:0_34:3 Triacylglycerols
TG 18:2_35:2 Triacylglycerols
TG 20:4_32:0 Triacylglycerols
TG 17:1_36:3 Triacylglycerols
TG 18:1_35:3 Triacylglycerols
TG 22:4_34:2 Triacylglycerols
LPI 18:0 Phosphatidylinositols
PI 18:0_18:0 Phosphatidylinositols
PI 17:1_18:1 Phosphatidylinositols
PA 18:1_18:1 Phosphatidic Acids
PG 16:0_16:0 Phosphatidylglycerols
PG 16:3_18:1 Phosphatidylglycerols
PC O-34:3 Phosphatidylcholines
PE P-16:0/15:(Phosphatidylethanolamines
PAG Aminoacids Related
ADMA Aminoacids Related
Ind-SO4 Indoles Derivatives
CE 22:5 Cholesterol Esters
p-Cresol-SO4 Cresols
Gly Aminoacids
HipAcid Carboxylic Acids
row min
row max
Aged
Mock
Mock
Mock
Mock
Mock
Infected
Infected
Infected
Infected
Infected
clusters
Metabolite
Class
C2 Acylcarnitines
Gln Aminoacids
1-Met-His Aminoacids Related
CE 20:4 Cholesterol Esters
CE 18:2 Cholesterol Esters
CE 16:0 Cholesterol Esters
PA 17:0_18:1 Phosphatidic Acids
PA 18:1_22:3 Phosphatidic Acids
PA 16:2_18:1 Phosphatidic Acids
PA 18:1_18:1 Phosphatidic Acids
PA 18:1_20:1 Phosphatidic Acids
PC O-36:5 Phosphatidylcholines
PC 36:4 Phosphatidylcholines
PC O-38:4 Phosphatidylcholines
PC O-40:4 Phosphatidylcholines
PC 36:0 Phosphatidylcholines
PC O-36:4 Phosphatidylcholines
PC 38:5 Phosphatidylcholines
PE 40:7 Phosphatidylethanolamines
PE 36:4 Phosphatidylethanolamines
PE 38:4 Phosphatidylethanolamines
PE 38:6 Phosphatidylethanolamines
PE 40:6 Phosphatidylethanolamines
PE 38:5 Phosphatidylethanolamines
PE 38:3 Phosphatidylethanolamines
PE 36:2 Phosphatidylethanolamines
PE 34:2 Phosphatidylethanolamines
PE 36:3 Phosphatidylethanolamines
PE 36:1 Phosphatidylethanolamines
LPE 16:0 Phosphatidylethanolamines
LPE 20:4 Phosphatidylethanolamines
LPE 18:0 Phosphatidylethanolamines
PE 38:7 Phosphatidylethanolamines
PE 38:0 Phosphatidylethanolamines
PE P-18:0/22:(Phosphatidylethanolamines
PE 36:0 Phosphatidylethanolamines
PE P-16:0/20:(Phosphatidylethanolamines
PE P-16:0/14:(Phosphatidylethanolamines
PE P-16:0/15:(Phosphatidylethanolamines
PG 18:1_22:5 Phosphatidylglycerols
PG 18:1_22:0 Phosphatidylglycerols
PG 17:1_18:1 Phosphatidylglycerols
LPI 18:0 Phosphatidylinositols
PI 18:1_20:2 Phosphatidylinositols
PI 16:0_18:2 Phosphatidylinositols
LPS 18:1 Phosphatidylserines
SM 40:4 Sphingomyelins
SM 38:3 Sphingomyelins
TG 20:4_36:3 Triacylglycerols
TG 20:4_32:0 Triacylglycerols
row min
row max
b
All triglycerides (µM)
2500
2000
1500
1000
500
0
*
mock
7 dpi
mock
7 dpi
Young
Aged
All phosphatidylethanolamines (µM)
25
20
15
10
5
0
**
mock
7 dpi
mock
7 dpi
Young
Aged

Supplementary Figure 9 – Influenza infection alters serum metabolomics differently in young mice and aged mice.

Serum samples from mock-treated and 7-dpi-infected young and aged mice were analyzed, n=5 per group. **(a)** Heatmaps showing differential metabolite levels between mock-treated and infected mice in both age groups ($P < 0.05$, linear model). Hierarchical clustering was performed by One Minus Pearson Correlation and Average linkage as the clustering method for young mice (88 metabolites) and aged mice (50 metabolites) to assess changes in metabolite profiles upon infection. Red/blue colors indicate increased/decreased levels. **(b)** Boxplots illustrating the average serum concentrations (μM) of the sum of all triglycerides (left) and phosphatidylethanolamines (right) in young and aged mice. Groups were compared using a two-sided Mann-Whitney test, with * indicating P values for mock vs. infected group comparisons ($^{*}P < 0.05$, $^{**}P < 0.01$). $P < 0.05$ was considered statistically significant.
