# Supplementary material for: Impact of aging on gut-lung-adipose tissue interactions and lipid metabolism during influenza infection in mice
Source: Sci Rep. 2025 Oct 27;15:37414. doi: 10.1038/s41598-025-21363-1 (PMC12559434; doi:10.1038/s41598-025-21363-1)
Supplement: Supplementary file 14 — Supplementary Information 14. [file 41598_2025_21363_MOESM14_ESM.pdf]

	Young			Aged		
	**Mock**	**7 dpi**	**28 dpi**	**mock**	**7 dpi**	**28 dpi**
Cells counted	15859	19587	15899	18432	18834	12392
Minimum	65.35	80.09	76.24	80.09	80.09	80.09
25% Percentile	295.37	210.15	345.34	243.15	262.37	269.74
Median	576.64	402.05	648.40	447.54	511.77	481.17
75% Percentile	971.32	710.55	1051.41	710.79	842.94	765.65
Maximum	5197.7	5828.24	6626.88	3407.95	4913.62	4108.89
Mean	709.63	525.91****	786.92****	520.70$^{\#\#\#}$	623.23****,	608.88****,
Std. Deviation	544.93	436.17	600.93	357.18	488.44	500.02
Std. Error of mean	4.33	3.12	4.77	2.63	3.56	4.49
Lower 95% CI of	701.15	519.80	777.58	515.55	616.26	600.08
Upper 95% CI of	718.11	532.01	796.26	525.86	630.21	617.68

Supplementary Table 1 – Descriptive statistics of adipocyte size frequency distribution in the SCAT.

The mean adipocyte sizes in the SCAT from young mice and aged mice at 0 (Mock) (n=4), 7 (n=4) and 28 (n=3) dpi were compared using a two-sided Mann-Whitney test. For intergroup differences, the threshold for statistical significance was set to $P < 0.05$. Values with superscripts symbols indicate significant differences (age group comparisons: $^{\#\#\#\#}$ $P < 0.0001$, and mock vs. infected group comparisons: **** $P < 0.0001$).
