# Supplementary material for: Impact of aging on gut-lung-adipose tissue interactions and lipid metabolism during influenza infection in mice
Source: Sci Rep. 2025 Oct 27;15:37414. doi: 10.1038/s41598-025-21363-1 (PMC12559434; doi:10.1038/s41598-025-21363-1)
Supplement: Supplementary file 15 — Supplementary Information 15. [file 41598_2025_21363_MOESM15_ESM.pdf]

	Young			Aged		
	**Mock**	**7 dpi**	**28 dpi**	**mock**	**7 dpi**	**28 dpi**
Cells counted	25161	18137	10628	16508	13735	7966
Minimum	80.09	65.35	80.09	80.09	44.53	80.09
25% Percentile	528.7	386.8	706.4	473.8	279.7	416.4
Median	1049	898.6	1503	1033	826.8	817.2
75% Percentile	1665	1613	2499	1770	1864	1385
Maximum	8382	8780	10394	8278	10709	8875
Mean	1187	1125^{****}	1755^{****}	1221	$1292^{****,\#}$	$1021^{****,\#\#\#\#}$
Std. Deviation	835.4	925.2	1340	921.2	1340	823.7
Std. Error of mean	5.267	6.870	12.99	7.169	11.43	9.229
Lower 95% CI of mean	1177	1112	1730	1207	1269	1003
Upper 95% CI of mean	1197	1139	1781	1235	1314	1039

Supplementary Table 2 – Descriptive statistics of adipocyte size frequency distribution in the VAT.

The mean adipocyte sizes in the VAT from young mice and aged mice at 0 (Mock) (n=7), 7 (n=7) and 28 (n=3) dpi were compared using a two-sided Mann-Whitney test. For intergroup differences, the threshold for statistical significance was set to $P < 0.05$. Values with superscripts symbols indicate significant differences (age group comparisons: $^{\#}P < 0.05$, $^{\#\#\#\#}P < 0.0001$), and mock vs. infected group comparisons: $^{****}P < 0.0001$).
