# Supplementary material for: Impact of aging on gut-lung-adipose tissue interactions and lipid metabolism during influenza infection in mice
Source: Sci Rep. 2025 Oct 27;15:37414. doi: 10.1038/s41598-025-21363-1 (PMC12559434; doi:10.1038/s41598-025-21363-1)
Supplement: Supplementary file 16 — Supplementary Information 16. [file 41598_2025_21363_MOESM16_ESM.pdf]

**Pathways**	**Role**	**Difference between medians**	***P*-value Welch correction**	***P*-value Wilcoxon correction**
FOLSYN.PWY	Tetrahydrofolate biosynthesis	-1.837093992	0.020912221	0.035828081
HEME.BIOSYNTHESIS.II	Heme biosynthesis (aerobic)	-1.919179035	0.020763524	0.034684302
HEMESYN2.PWY	Heme biosynthesis (anaerobic)	-1.944476933	0.020763484	0.039256911
PWY.3781	Aerobic respiration (cytochrom c)	-1.689696246	0.034113122	0.037207883
PWY.5918	Heme b biosynthesis	-1.935305324	0.020749447	0.034644294
PWY.6612	Tetrahydrofolate biosynthesis	-1.888003463	0.020761408	0.036014789
PWY0.845	Pyridoxal 5'-phosphate biosynthesis	-1.938117716	0.020937935	0.034644294
PYRIDOXSYN.PWY	Pyridoxal 5'-phosphate biosynthesis	-1.822626212	0.024149999	0.035939154

Supplementary Table 4 – Age-associated alterations of gut microbial function.

Eight MetaCyc pathways showed significant ($P < 0.05$) changes in young vs. aged mice. Seven pertained to cofactor, carrier, and vitamin biosynthesis, and one related to metabolite precursor generation and mitochondrial electron transfer chains.
