# Supplementary material for: Impact of aging on gut-lung-adipose tissue interactions and lipid metabolism during influenza infection in mice
Source: Sci Rep. 2025 Oct 27;15:37414. doi: 10.1038/s41598-025-21363-1 (PMC12559434; doi:10.1038/s41598-025-21363-1)
Supplement: Supplementary file 17 — Supplementary Information 17. [file 41598_2025_21363_MOESM17_ESM.pdf]

Groups	Total	KEGG pathways
Increased at 4, 7 and 28 dpi	18	Parkinson disease<br>Electron transfer carriers<br>Various types of N glycan biosynthesis<br>Metabolism of xenobiotics by cytochrome P450<br>Influenza A<br>Naphthalene degradation<br>Colorectal cancer<br>p53 signaling pathway<br>Toxoplasmosis<br>Viral myocarditis<br>Drug metabolism cytochrome P450<br>Phenylalanine metabolism<br>Small cell lung cancer<br>Glycan biosynthesis and metabolism<br>Tyrosine metabolism<br>Protein processing in endoplasmic reticulum<br>Alzheimer s disease<br>Meiosis yeast
Increased at 4 and 7 dpi	8	Bacterial motility proteins<br>Amyotrophic lateral sclerosis<br>Sulfur metabolism<br>Carbon fixation pathways<br>Biosynthesis and biodegradation of secondary metabolites<br>Flagellar assembly<br>Pathways in cancer<br>Fatty acid metabolism
Increased at 7 and 28 dpi	8	Adipocytokine signaling pathway<br>Nitrotoluene degradation<br>Lipopolysaccharide biosynthesis proteins<br>Lipopolysaccharide biosynthesis<br>Protein folding and associated processing<br>Apoptosis<br>Retinol metabolism<br>Lipoic acid metabolism
Increased only at 4 dpi	5	Tuberculosis<br>Methane metabolism<br>Bacterial chemotaxis<br>Taurine and hypotaurine metabolism<br>Two component system
Increased only at 7 dpi	4	Citrate cycle (aka TCA cycle)<br>Ubiquinone and other terpenoid quinone biosynthesis<br>Mineral absorption<br>Aminobenzoate degradation

Supplementary Table 5 – Increased KEGG functional pathways in influenza-infected young gut microbiota.

KEGG pathways significantly increased ($P < 0.05$) upon infection at 4, 7, 14 and 28 dpi in the gut microbiota of young mice (lists of shared or specific pathways at different post-infection time points are shown).
