# Supplementary material for: Impact of aging on gut-lung-adipose tissue interactions and lipid metabolism during influenza infection in mice
Source: Sci Rep. 2025 Oct 27;15:37414. doi: 10.1038/s41598-025-21363-1 (PMC12559434; doi:10.1038/s41598-025-21363-1)
Supplement: Supplementary file 18 — Supplementary Information 18. [file 41598_2025_21363_MOESM18_ESM.pdf]

Groups	Total	KEGG pathways
Decreased at 4, 7, 14 and 28 dpi	1	Glycosphingolipid biosynthesis
Decreased at 4, 7 and 14 dpi	2	Other glycan degradation<br>Steroid hormone biosynthesis
Decreased at 4, 7 and 28 dpi	1	Steroid hormone biosynthesis
Decreased at 4 and 7 dpi	4	Glycosphingolipid biosynthesis<br>Glycosaminoglycan degradation<br>Lysosome<br>Carotenoid biosynthesis
Decreased at 4 and 28 dpi	1	Ion channels
Decreased at 7 and 28 dpi	1	Transcription related proteins
Decreased only at 4 dpi	2	Flavone and flavonol biosynthesis<br>Siderophore group biosynthesis
Decreased only at 28 dpi	2	Staphylococcus aureus infection<br>Ether lipid metabolism

Supplementary Table 6 – Decreased KEGG functional pathways in influenza-infected young gut microbiota.

KEGG pathways significantly decreased ($P < 0.05$) upon infection at 4, 7, 14 and 28 dpi in the gut microbiota of young mice (lists of shared or specific pathways at different post-infection time points are shown).
