# Supplementary material for: Impact of aging on gut-lung-adipose tissue interactions and lipid metabolism during influenza infection in mice
Source: Sci Rep. 2025 Oct 27;15:37414. doi: 10.1038/s41598-025-21363-1 (PMC12559434; doi:10.1038/s41598-025-21363-1)
Supplement: Supplementary file 19 — Supplementary Information 19. [file 41598_2025_21363_MOESM19_ESM.pdf]

Groups	Total	Lipids
Both in young and aged mice	5	TG 20:4_36:3, LPI 18:0, PA 18:1_18:1<br>PE P-16:0/15:0, TG 20:4_32:0
Only in young mice	77	TG 16:0_34:4, TG 20:1_34:3, TG 18:1_36:0<br>TG 16:0_36:3, TG 16:0_34:2, TG 18:1_32:0<br>TG 16:0_36:5, TG 18:1_34:1, TG 16:0_34:1<br>TG 16:0_36:4, TG 16:0_38:5, TG 22:4_34:2<br>PG 16:0_16:0, TG 22:6_32:0, TG 16:0_34:3<br>TG 18:0_34:3, TG 16:0_35:2, TG 18:2_38:6<br>TG 18:1_35:3, TG 18:2_36:2, TG 18:1_38:5<br>TG 16:0_32:1, TG 18:3_32:1, TG 18:1_36:5<br>PG 16:3_18:1, TG 18:2_36:3, TG 18:2_38:5<br>TG 20:4_34:2, TG 18:1_36:3, CE 22:5<br>TG 16:0_36:2, TG 20:2_34:1, TG 22:6_32:1<br>TG 16:0_32:2, PI 17:1_18:1, TG 18:2_32:1<br>TG 20:5_34:1, TG 16:1_38:5, TG 18:2_35:2<br>TG 18:2_34:3, TG 16:1_34:2, TG 18:2_32:2<br>TG 16:1_36:3, TG 18:1_32:3, PC O-34:3<br>TG 20:4_34:1, TG 18:1_34:2, TG 20:4_36:4<br>TG 18:1_32:1, TG 16:1_36:4, TG 16:1_32:1<br>TG 18:1_38:6, TG 16:0_38:4, TG 22:6_34:1<br>TG 18:0_36:4, TG 16:1_34:1, TG 18:3_34:1<br>TG 18:1_38:7, TG 17:1_34:2, TG 20:4_32:1<br>TG 18:2_34:2, TG 18:1_32:2, TG 16:0_38:6<br>TG 18:3_34:2, TG 16:1_34:3, TG 18:0_36:3<br>TG 17:1_36:3, TG 18:2_34:1, PI 18:0_18:0<br>TG 16:0_35:3, TG 18:2_36:4, TG 18:1_34:3<br>TG 20:4_36:2, TG 14:0_36:2, TG 16:1_36:2<br>TG 20:3_34:2, TG 20:4_34:3
Only in aged mice	43	PC 36:4, PE 38:6, PA 18:1_20:1, PE 36:4<br>PE 36:3, CE 18:2, PC O-38:4, C2, PA 18:1_22:3<br>PE P-16:0/20:4, PI 16:0_18:2, PE 34:2, PE 36:0<br>LPE 18:0, LPS 18:1, CE 20:4, PC O-36:5<br>PE 38:0, PC 38:5, PA 17:0_18:1, PE 38:4<br>PE 38:7, PE 40:6, LPE 20:4, PC 36:0<br>PE P-16:0/14:0, PE 36:2, PC O-36:4, PE 38:5<br>PG 17:1_18:1, PE P-18:0/22:6, LPE 16:0<br>SM 40:4, PA 16:2_18:1, PI 18:1_20:2, PC O-40:4<br>CE 16:0, PE 40:7, PG 18:1_22:0, SM 38:3<br>PG 18:1_22:5, PE 38:3, PE 36:1

Supplementary Table 7 – Shared and specific serum lipids modulated by influenza infection in young and aged mice.

Lists of the lipids significantly changed upon infection in young mice only (77 lipids), in both young and aged mice (5 lipids), and in aged mice only (43 lipids).
