# Supplementary material for: Impact of aging on gut-lung-adipose tissue interactions and lipid metabolism during influenza infection in mice
Source: Sci Rep. 2025 Oct 27;15:37414. doi: 10.1038/s41598-025-21363-1 (PMC12559434; doi:10.1038/s41598-025-21363-1)
Supplement: Supplementary file 20 — Supplementary Information 20. [file 41598_2025_21363_MOESM20_ESM.pdf]

Genes		Sequences
Gapdh	Forward	5’ GCAAAGTGGAGATTGTTGCCA 3’
	Reverse	5’ GCCTTGACTGTGCCGTTGA 3’
Eef2	Forward	5’ CACAACATGCGCGTCATGA 3’
	Reverse	5’ AGACTTAGCCAGCCGCTTCAG 3’
Cd3e	Forward	5’ AACACGTACTTGTACCTGAAAGCTC 3’
	Reverse	5’ GATGATTATGGCTACTGCTGTCA 3’
Cd8a	Forward	5’ CGTGTCCCTCATGGCAGAA 3’
	Reverse	5’ AACAAGATAACGTGGGACGAGAA 3’
Cd4	Forward	5’ CCTTAGGTTGGACATGGAGAGAA 3’
	Reverse	5’ CCCCTTGACAGAGTGCAAACA 3’
Tnfa	Forward	5’ ACGGCATGGATCTCAAAGAC 3’
	Reverse	5’ AGATAGCAAATCGGCTGACG 3’
Il1b	Forward	5’ TTGACGGACCCCAAAAGATG 3’
	Reverse	5’ AGAAGGTGCTCATGTCCTCA 3’
Il6	Forward	5’ AGCCTCCGACTTGTGAAGTG 3’
	Reverse	5’ CTGATGCTGGTGACAACCAC 3’
Il10	Forward	5’ CCAGTCGGCCAGAGCCACAT 3’
	Reverse	5’ GCTTCTCTGCCTGGGGCATC 3’
Cd11b	Forward	5’ CCATGACCTTCCAAGAGAATGC 3’
	Reverse	5’ ACCGGCTTGTGCTGTAGTC 3’
RigI	Forward	5’ TGCGGAAATACAACGATGCA 3’
	Reverse	5’ GCTCGGTCTCATCGAATGCTG 3’
Mda5	Forward	5’ TGATGCACTATTCCAAGAACTAACA 3’
	Reverse	5’ TCTGTGAGACGAGTTAGCCAAG 3’
Mx1	Forward	5’ TGCAGAGGTCAGCAGGACATC 3’
	Reverse	5’ GGCAGTTTGGACCATCTCTGAA 3’
Cd11c	Forward	5’ CCTGAGGGTGGGCTGGAT 3’
	Reverse	5’ GAAGAGGCGGCAGCAGATCG 3’
Isg20	Forward	5’ ACAAGTACATCCGACCCGAGG 3’
	Reverse	5’ GCCTTTCAGAAGCTGCAGGA 3’
Lipe	Forward	5’ GCATTGTGCCCTGCTCGGTT 3’
	Reverse	5’ CAGTGACGCAGAGGTTGCCG 3’
Pnpla2(Atgl)	Forward	5’ GGCCTACTGAACCAACCCAA 3’
	Reverse	5’ GAAGGCAGATGGTCACCCAA 3’
Mgl	Forward	5’ AATCCGGAATCTGCATCGACT 3’
	Reverse	5’ AACCTCCGACTTGTTCCGAGAC 3’
Glut4	Forward	5’ AGGGCTGCAAAGCGTAGGTA 3’
	Reverse	5’ TGGTCAGAAGCTTTCGGGTTT 3’
Ucp1	Forward	5’ GCTTTGCCTCACTCAGGATTGG 3’
	Reverse	5’ CCAATGAACACTGCCACACCTC 3’

Supplementary Table 8 –– Sequences of the forward and reverse primers.

Primers were designed using the Primer Express^{TM} v3 software. *Gapdh*: glyceraldehyde 3-phosphate dehydrogenase, *Eef2*: eukaryotic translation elongation factor 2, *Cd(3e-4-8a-11b-11c)*: cluster of differentiation, *Tnfa*: tumor necrosis factor alpha, *Il1b*: interleukin-1 beta, *Il10*: interleukin-10, *Il6*: inteleukin-6, *RigI*: retinoic acid-inducible gene I, *Mda5*: melanoma differentiation-associated protein 5, *Mx1*: Mx dynamin like GTPase 1, *Isg20*: interferon stimulated exonuclease gene 20, *Lipe*: lipase E, *Pnpla2* (Atgl): patatin-like phospholipase domain containing 2, *Mgl*: monoglyceride lipase, *Glut4*: glucose transporter type 4, *Ucp1*: uncoupling protein 1.
